# Calibration of a fully closed-loop lumped-parameter cardiovascular model with baroreflex in response to induced central hypovolemia

**DOI:** 10.1371/journal.pone.0362073

**Published:** 2026-11-06

**Authors:** Karolina Tlałka, Harry Saxton, Ian Halliday, Xu Xu, Andrew Narracott, Daniel James Taylor, Lars Øivind Høiseth, Jonny Hisdal, David Rodney Hose, Maciej Malawski

**Affiliations:** 1 Division of Clinical Medicine, School of Medicine and Population Health, University of Sheffield, Sheffield, United Kingdom; 2 Sano Centre for Computational Medicine, Kraków, Poland; 3 Astellas Pharma Europe Ltd, Addlestone, England; 4 Insigneo Institute for Healthcare Technology, Sheffield, United Kingdom; 5 School of Computer Science, University of Sheffield, Sheffield, United Kingdom; 6 NIHR Sheffield Biomedical Research Centre, Sheffield Teaching Hospitals NHS Foundation Trust, Sheffield, United Kingdom; 7 Department of Anesthesia and Intensive Care Medicine, Division of Emergencies and Critical Care, Oslo University Hospital, Oslo, Norway; 8 Department of Vascular Surgery, Division of Cardiovascular and Pulmonary Diseases, Oslo University Hospital, Oslo, Norway; 9 Institute of Clinical Medicine, Faculty of Medicine, University of Oslo, Oslo, Norway; 10 Faculty of Computer Science, AGH University of Kraków, Kraków, Poland; University of Missouri, UNITED STATES OF AMERICA

## Abstract

Calibration of cardiovascular models is essential to their clinical utility. We provide a full description of the calibration of a non-linear, dynamic model using global optimisation methods. Specifically, we consider an experimental cohort response in a closed-loop, baro-regulated cardiovascular model, in the setting of induced central hypovolemia.We propose a framework for simplifying calibration of dynamic, regulated models. We perform global sensitivity analysis and orthogonality analysis of a 58-parameter non-linear cardiovascular model including the baroreflex, to select an optimal subset of parameters for model calibration. We fit the model to relative Lower Body Negative Pressure data, using global optimisation (Differential Evolution method), searching a reduced input parameter space. Global sensitivity analysis and orthogonality analysis revealed multiple higher-order interactions within the model, as well as areas of similar influence of input parameters, on the components of the objective function. The model was fitted using only baroreflex parameters. Relative errors in the estimation of heart rate, mean arterial pressure, and stroke volume were reduced from −15.03%, 1.60% and 42.06% before calibration to 0.42%, −1.37% and 0.61% after the fitting, respectively.

## Introduction

According to the World Health Organisation (WHO), cardiovascular disease is the leading cause of death, claiming 17.9 million lives each year [1]. Such numbers pose a significant challenge for healthcare systems worldwide. By representing human cardiovascular (patho)physiology, physics-informed mathematical modeling supports prevention (e.g., by estimating the risk of disease occurrence or progression [2]), and diagnosis (e.g., by replacing invasive procedures or optimising hemodynamic prosthesis design- see Morris et al. and references therein [3]) – if such models can first be calibrated to individual or cohort data. Under such circumstances, models might serve as a digital representation of a subject or a cohort, facilitating calibrated, more efficient healthcare [4].

In operation the cardiovascular system is continuously perturbed by environmental challenges- such as orthostatic, gravitational, emotional and physical stress (exercise) *inter alia* [5–7]. Integrating mechanical cardiovascular models with homeostatic regulation models is therefore essential to enhance physiological fidelity and model applicability. Homeostasis is maintained by hormonal and neural negative feedback mechanisms, most importantly the baroreflex [8]. The latter regulates arterial pressure over short timescales (seconds to minutes), modulating both cardiac function (heart rate and contractility) and vascular properties (peripheral resistance and venous tone) [9]. Baroreflex sensitivity – a key clinical indicator of baroreflex function – varies across individuals and is strongly influenced by, e.g., age and sex [10].

The baroreflex maintains arterial pressure during any challenge [5,11]. Abnormal function may lead to orthostatic intolerance (orthostatic hypotension or hypertension) [12]. There are several experimental tests which have been used to examine these effects of which lower body negative pressure (LBNP) is the simplest. This is an experiment in which the blood volume of a supine subject is rapidly *misallocated* away from the upper body to the legs by reducing the pressure on the lower body. This leads to central hypovolemia, which causes multiple changes in blood flow and respiration [13], reducing both venous return and circulating blood volume, disrupting cardiovascular function, and affecting clinical metrics such as cardiac output (CO), stroke volume (SV) and mean arterial pressure (MAP). A drop in MAP activates the neurohumoral baroreflex response, driven primarily by the sympathetic nervous system. When hypovolemia persists for more than about 10 minutes the renin–angiotensin system is also activated, which contributes further to circulatory regulation [13]. Typically this same effect may occur during gravitational stress, for example during spaceflight [14] or during simple tilt table tests [5].

In 2024, Morris et al.[15], reviewed mathematical models of orthostatic stress response. These authors’ synoptic Table 1 [15] summarises relevant work from as early as 1974 [16]; from this *prior art* the contributions most relevant to our work are as follows. Melchior et al. [17] developed a computational cardiovascular system model to interpret data on LBNP response, with particular reference to spaceflight physiology, using a remarkably straightforward lumped-parameter framework, accounting for blood volume redistribution, reduced left ventricular filling (decreased cardiac preload), heart–vascular system interaction and primitive baroreflex control of the heart rate and vascular resistance. It also predicts qualitative trends in orthostatic intolerance, spaceflight countermeasure effects and LBNP response in autonomic blockade. All subsequent work -including ours- uses these same elements. Later, Heldt et al. applied a lumped parameter model to head up tilt (HUT) responses which assumes a constant point of indifference [18] and advances the baroreflex and cardio-pulmonary reflex representation, based on integral control [5]. Most recently, Fois at al [19]., presented a multi-scale computational model to simulate cardiovascular responses to HUT, predicting changes in blood pressure and heart rate validated on *in vivo* HUT tests.

**Table 1:** Mechanical sub-model input parameters of the model declared diagrammatically in Fig 2, adjusted based on Ursino [9]. The constant values declared in the table above we designate base values. Regulated input parameters are modulated by the appropriate regulation effector [9]. The parameterisation of our regulation sub-model are to be found in Table 3, reference [9]. Parameters marked with * are regulated.

Parameter	Symbol	Value	Unit
Mean circulatory filling pressure	*MCFP*	7.00	*mmHg*
Maximum left atrial elastance	$E_{LAmax}$	0.50	$\frac{mmHg}{ml}$
Minimum left atrial elastance	$E_{LAmin}$	0.27	$\frac{mmHg}{ml}$
Initial maximum left ventricular elastance*	$E_{LVmax,0}$	1.75	$\frac{mmHg}{ml}$
Minimum left ventricular elastance	$E_{LVmin}$	0.10	$\frac{mmHg}{ml}$
Maximum right atrial elastance	$E_{RAmax}$	0.24	$\frac{mmHg}{ml}$
Minimum right atrial elastance	$E_{RAmin}$	0.12	$\frac{mmHg}{ml}$
Initial maximum right ventricular elastance*	$E_{RVmax,0}$	0.52	$\frac{mmHg}{ml}$
Minimum right ventricular elastance	$E_{RVmin}$	0.06	$\frac{mmHg}{ml}$
Initial heart period*	$\tau_{HR,0}$	0.83	*s*
Time of the initiation of systolic phrase (atria)	$\tau_{initA}$	0.90 $\tau_{HR}$	*s*
Time of the initiation of systolic phrase (ventricles)	$\tau_{initV}$	0.00 $\tau_{HR}$	*s*
Heart valves resistance	$r_{mv},r_{av},r_{tv},r_{pv}$	0.005	$\frac{mmHg\cdot s}{ml}$
Aortic compliance	$C_{Ao}$	0.28	$\frac{ml}{mmHg}$
Aortic resistance	$R_{Ao}$	0.01	$\frac{mmHg\cdot s}{ml}$
Upper body arterial compliance	$C_{sA}$	2.05	$\frac{ml}{mmHg}$
Upper body arterial resistance*	$R_{sA}$	2.00	$\frac{mmHg\cdot s}{ml}$
Upper body venous compliance*	$C_{sV}$	61.11	$\frac{ml}{mmHg}$
Upper body venous resistance	$R_{sV}$	0.038	$\frac{mmHg\cdot s}{ml}$
Common iliac artery resistance	$R_{Ao2}$	0.001	$\frac{mmHg\cdot s}{ml}$
Lower body arterial compliance	$C_{eA}$	1.67	$\frac{ml}{mmHg}$
Lower body arterial resistance*	$R_{eA}$	1.50	$\frac{mmHg\cdot s}{ml}$
Lower body venous compliance*	$C_{eV}$	50.00	$\frac{ml}{mmHg}$
Lower body venous resistance	$r_{eV}$	0.016	$\frac{mmHg\cdot s}{ml}$
Pulmonary arterial compliance	$C_{pulA}$	5.80	$\frac{ml}{mmHg}$
Pulmonary arterial resistance	$R_{pulA}$	0.04	$\frac{mmHg\cdot s}{ml}$
Pulmonary venous compliance	$C_{pulV}$	25.37	$\frac{ml}{mmHg}$
Pulmonary venous resistance	$R_{pulV}$	0.0056	$\frac{mmHg\cdot s}{ml}$
Maximum venous volume	$V_{max}$	1000.00	ml

We aim to identify a closed-loop, baroregulated model, simpler than previously published ([5,17]), and to establish the means to tune it to central cardiovascular perturbations associated with an LBNP challenge, using data from a cohort of subjects [20]. We reproduce reported hemodynamic disturbances (in stroke volume, heart rate and mean arterial pressure), using the combination of a widely cited baroreflex model [9] and a simple cardiovascular model, demonstrating that it is possible to achieve good fits to experimental data with a model which is significantly less structurally complex than those in the current *prior art* [5,21]- at least in the present setting. Our secondary aim is to provide a mechanistic framework for interpreting differences in a dynamic cardiovascular response, to a controlled, orthostatic perturbation, rather than to extract baroreflex parameters and to use them for phenotyping, or as biomarkers. Potential clinical applications of our work involve analysing experimental protocols, in which we find the subset of model input parameters for a specific subject under small magnitude LBNP, and then use the calibrated subset to predict this subject’s percentage change of experimental data in response to higher magnitude LBNP, which could potentially generate the risk if applied *in vivo*.

### Background

The zero-dimensional (0D) model proposed by Ursino [9] provides a popular representation of baroreflex control, explicitly describing the afferent and efferent paths and principal effectors, to reflect essential physiology. It has been used widely and extended by several authors [21–23]. Some more complex models exist. Schild proposed a representation of the afferent element, based on Hodgkin-Huxley type membranes [24], which was later used by Sturdy et al. [25] and Fernandes et al. [8]. Sturdy et al. developed the most detailed model of afferent signal generation, in which every stage of receptor firing is represented using non-linear sigmoidal functions for arterial wall deformation, two Voigt bodies with springs for nerve ending deformation, the Boltzmann relationship for the fraction of opened mechanosensitive ion channels and neuronal modeling for action potential generation. See Sturdy [25] and the references therein. Fernandes et al. [8] proposed a more uniform solution, which incorporates a Hodgkin-Huxley [24,26] approach for all parts of the afferent processing (baroreceptor, nerve fiber, synaptic terminal and NTS neurons), and coupled it with the Danielsen and Ottesen model [27], for efferent processing [8]. Mahdi et al. [28] developed a systematic modeling framework for the afferent dynamics of baroreflex control, demonstrating that non-linear wall deformation properties are essential for accurate pressure-firing rate relationships and that integrate-and-fire models are necessary to capture post-excitatory depression. Beyond single-system approaches, several groups have developed integrated cardio-respiratory models that incorporate baroreflex control alongside other regulatory mechanisms (Sarmiento et al. [29], Van Willigen et al. [30]). The complex integrated approach poses significant challenges compared to simpler cardiac or baroreflex-only models, as it requires coupling representations of multiple physiological systems, operating on different characteristic time scales. Both detailed mechanistic studies ([8,25]) and integrated system approaches ([29,30]) provide deep insight into cardiovascular regulation physiology, but their complexity makes them unsuitable in closed-loop operation alongside mechanical models for real-time applications.

Calibration refers to the process of estimating model parameters, based on subject-specific data, with the goal of aligning the model output to the observed physiological response [31]. A standard approach to personalising cardiovascular models with baroreflex control involves solving this inverse problem, typically using a least squares method to minimise the error between experimental data and model predictions [21,32,33]. In complex models, calibration (or optimisation) is often performed on a subset of influential or sensitive input parameters, to reduce computational cost. The identification of such input parameter subsets relies on sensitivity analyses, which expose the most influential input parameters on model results. Sensitivity analysis may be followed by further refinement of the parameter subset, using methods such as structural covariance analysis [34], singular value decomposition with QR factorisation [34,35], multi-start inference studies ([36,37]), subspace selection [34], or -as here- orthogonality analysis [38].

There are several works describing the calibration of baroregulated models. Lim et al. used a zero-dimensional cardiovascular model with ventricular interaction, coupled with Ursino’s [9] reflex model, to simulate responses to a graded head-up tilt [21], identifying the point of indifference at the coronaries. They performed a relative local sensitivity analysis to determine the most influential parameters. Dependent parameters were identified using the covariance matrix. The subset of optimisation parameters was then systematically reduced through multiple iterations of gradient-based, physiologically constrained optimisation, excluding the most covariant parameter at each step [21]. In contrast, Olufsen et al. [32] avoided the need to solve a constrained optimisation problem by parameterising variables with predefined upper and lower bounds. Unlike Lim et al.’s gradient-based approach [21], they applied a sampling-based Nelder-Mead method, to calibrate the response of their baroreflex model (modulating only heart rate) to a sit-to-stand test in an open-loop setting [32]. Ottesen et al. employed their structural correlation method to identify the least sensitive and correlated parameters, which were fixed during Levenberg-Marquardt gradient-based optimisation to calibrate to a head-up tilt test response [31]. Matzuka et al. took a different approach by estimating time-varying parameters during head-up tilt, comparing piecewise linear splines with the ensemble transform Kalman filter (ETKF), to predict dynamic changes in cardiac contractility and vascular resistance [39]. The ETKF’s recursive state estimation approach also accounts for model error, acknowledging the limitations of simplified lumped-parameter models. The Valsalva maneuver has also attracted attention. Kosinski et al. [33] used least squares with a gradient method to fit two baroreflex response models to experimental data- a simple linear filter model, and a more complex physiologically-based model. A notable finding was that model complexity was not necessarily correlated with fit quality [33].

### Study rationale

Adaptability is one of the most important factors determining the suitability of a model as a clinical digital twin; it is therefore a crucial consideration in their development. The novelty of the paper lies in the aspects listed below:

1. Using a fully global framework to characterise and calibrate the model. Most *prior art* uses local sensitivity analysis to analyse models with a baroreflex [21,31,34]. As Ottesen and Olufsen realised [34], when the uncertainty of input parameters is high, global methods may be of additional value to the analysis, as they are independent of initial input estimates. Here, we analyse a cardiovascular model with systemic and pulmonary circulations, which is regulated, i.e., its input parameters are constantly adjusted by a coupled baroreflex regulation model [9,40] using global methods only. We propose a method to calibrate a dynamic cardiovascular model which utilises loss-function-oriented global sensitivity analysis, orthogonality analysis and adapted Differential Evolution. Based on optimisation-orientated global sensitivity analysis and orthogonality analysis, we identify the subset of input parameters for calibration, and then use modified Differential Evolution (DE) optimiser to fit the model response to chosen experimental data.
2. Using modified Differential Evolution to calibrate the model. Most authors use methods suited for locating a local minimum of the loss function – gradient-based [33] or heuristic [32] to calibrate cardiovascular models with baroreflex. We devise a practical method for addressing convergence problems in global optimisation, (Fig 1); our approach is based on adapting the resolution of our DE search.
3. Model simplicity. Most *prior art* uses relatively complex models to simulate LBNP [5,21] with architecturally complex systemic circulations, including leg, arm, upper body etc. [5] compartments. Here, we perform the first global calibration of a much simpler zero-dimensional vascular model (pulmonary, lower body and upper body compartments only), coupled with the regulation model of Ursino [9]. While a focus of our work is to develop methodology adequate to address complex coupled models applied to time-distributed challenges, our framework is based on a particular simpler example. That said, our work compliments *prior art* utilizing more detailed mechanical sub-models, which, in contrast to our model, are designed to resolve detailed blood volume shifts [5], but are very hard to calibrate due to the number of parameters.
4. Accounting for the non-linear properties of venous compliance. To the best of our knowledge, this aspect have not been considered in any *prior art* associated with calibrated LBNP models, but we believe, that it is essential in any model setting where blood volume misallocation is important.

**Fig 1:**
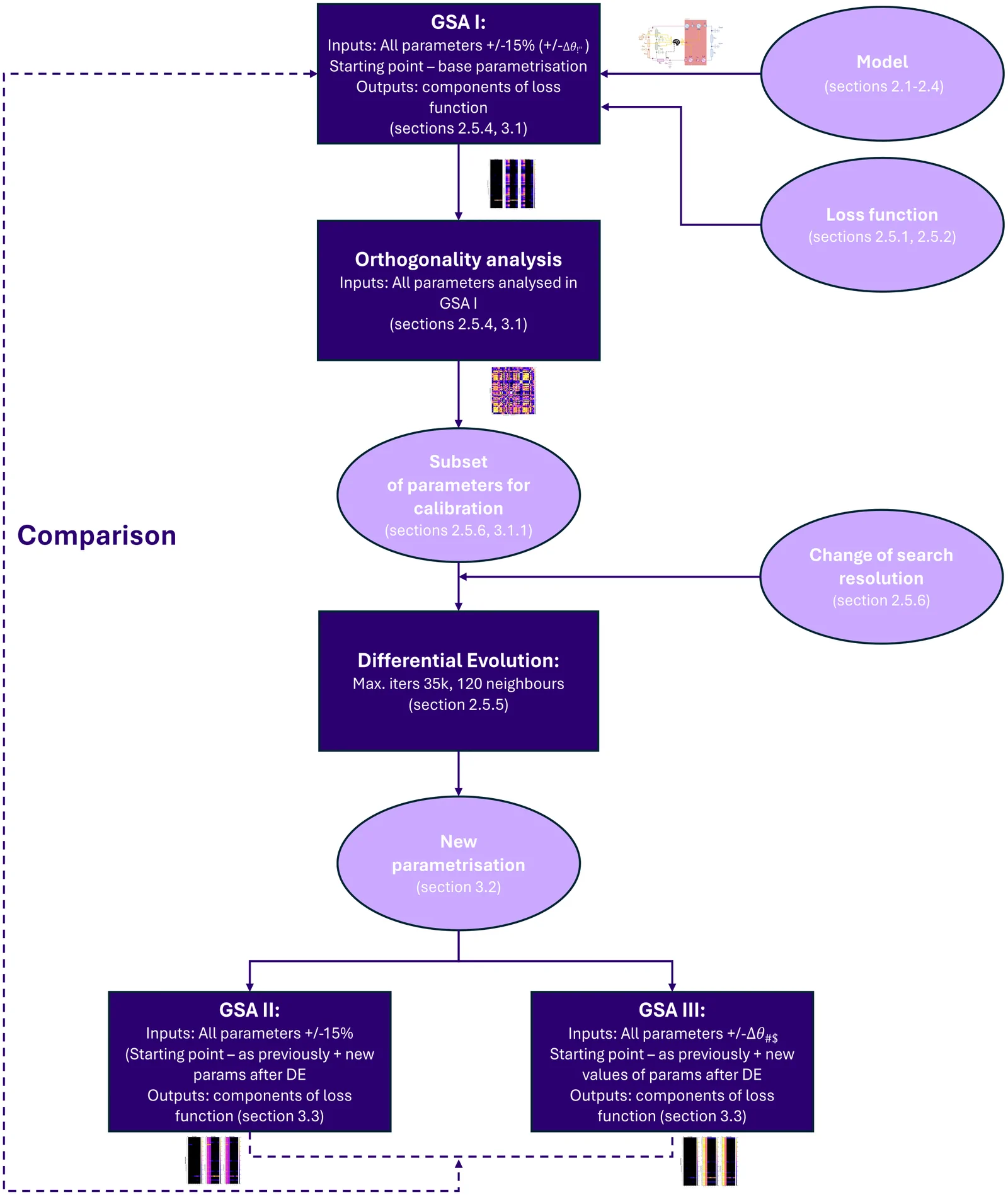
A schematic of our method. After identifying the model and loss function, we conduct global sensitivity analysis (GSA) over a ±15% range, with components of our loss function as the outputs. Based on the results of GSA, tuning requirements and orthogonality analysis, we identify a subset of parameters for model calibration, having wide variation (i.e., uncertainty). We vary the resolution of the search, depending on the magnitude of each input parameter. Then, over a reduced volume of input parameter space, we perform global optimisation, which is repeated 100 times, to take into account possible misleading searches resulting from rounding, and choose the solution with the smallest value of the objective function. We repeat the GSA, varying input parameters over the same percentage bands as previously (GSA II) or varying input parameters by the same absolute change as previously (GSA III), now starting from the point identified during global optimisation, to check how the sensitivity pattern differs, compared to that at base state.

This work, then, leverages our sensitivity analyses of unperturbed (subject to no external stimuli), closed-loop cardiovascular models of varied structural complexity [40]. The present study extends:

- the cardiovascular model complexity (previously, we analysed one and elementary four-chamber models, here we treat a more complex systemic circulation architecture);
- the scope of the model (previously, we reported model sensitivity and orthogonality, here we apply previous findings in an end-to-end model calibration);
- the modeling framework (here we consider sensitivities based on a loss function and devise the means to treat an external stimulus – LBNP).

## Materials and methods

This section provides an overview of our research techniques, from coupled model development (synthesis of mechanical and existing baroreflex sub-models, and reduced-order modeling), the coupled model setting (description of models and their respective stimuli), through model quantification (GSA), reduction of the input parameter volume (grid searching and adapting the spatial resolution) and finally, calibration to experimental data (using DE [41] optimisation).

### Mechanical sub-model

Here, we utilise a zero-dimensional approach. Electrical analogue formulations of cardiovascular dynamics map electrical quantities of current and voltage to flow and pressure and resistors, diodes and capacitance to hemodynamic dissipation, valves and vessel compliance respectively [42]. The state variables – pressure ($P_{k}$), flow ($Q_{k}$) and volume ($V_{k}$) – of the *k*-th passive compartment are related using the following equations [40]:


$$\begin{array}{c} \frac{dP_{k}}{dt}=\frac{1}{C_{k}}(Q_{k}-Q_{k+1}), \end{array}$$ (1)

$$\begin{array}{c} Q_{k}=\frac{P_{k-1}-P_{k}}{R_{k}}, \end{array}$$ (2)

$$\begin{array}{c} \frac{dV_{k}}{dt}=Q_{k}-Q_{k+1}. \end{array}$$ (3)

Above, $C_{k}$, $R_{k}$ represent the compliance and resistance of the k-th component. The equations above may be applied to all the compartments and the flow variables eliminated, to obtain a state-space formulation [43] for the system in terms of compartmental pressures only. This process is straightforward, with the exception of the dynamics of the venous compartment, which interacts strongly with the system regulation and is considered in section *LBNP orthostatic challenge and unstressed volume regulation*.

As evidenced in Equation (3), stressed volume $V_{k}$ (defined as the volume of fluid above the level of unstressed vessel filling [44]) can also be calculated, as the integral of net flow across the compartment boundary. The total volume $V_{T,k}$ is calculated as the sum of stressed $V_{k}$ and unstressed $V_{U,k}$ volumes. Model equations can be formulated in terms of pressure (Equation (1)) or volume (Equation (3)); these formulations are equivalent.

The representation of active components follows our previous work [40]. Heart chamber function is represented by a time- varying elastance, $E_{i,V}$, following Suga [45], using Korakianitis and Shi’s [46] approach, with an activation function, *e*(*t*) defined by gradient-continuous, piecewise trigonome*t*ric functions, with only two time constants, one for the peak of the systolic phase ($\tau_{es}$), the other for the end of the systolic phase ($\tau_{ss}$). For the atria, an additional time shift $\tau_{init}$ is introduced, to accommodate the phase offset between the start of atrial and ventricular systole [40]:


$$\begin{array}{c} E_{iA}(t)=(E_{iA,max}-E_{iA,min})\cdot e_{A}(t)+E_{iA,min}, \end{array}$$ (4)

where


$$\begin{array}{c} e_{A}(t)=\left\{ \begin{array}{cc} 0, & 0\leq t<\tau_{init},\\ \frac{1}{2}[1-\cos\left(\frac{\pi (t-\tau_{init})}{\tau_{iA,es}-\tau_{init}}\right)], & \tau_{init}\leq t<\tau_{iA,es},\\ \frac{1}{2}[1+\cos\left(\frac{\pi (t-\tau_{iA,es})}{\tau_{iA,ss}-\tau_{iA,es}}\right)], & \tau_{iA,es}\leq t<\tau_{iA,ss},\\ 0, & \tau_{iA,ss}\leq t<\tau_{HR}. \end{array}\right. \end{array}$$ (5)

Time constants were fixed as a constant proportion of the heart cycle: for ventricles, the time of the peak of the systolic part $\tau_{iV,es}=0.30\tau_{HR}$ and the time of the end of systolic phrase $\tau_{iV,ss}=0.45\tau_{HR}$; for the atria $\tau_{iA,es}=0.96\tau_{HR}$ and $\tau_{iA,ss}=1.00\tau_{HR}$.

Valves are modeled as simple diodes, with a high resistance value $r_{iv}$ under retrograde flow and relatively small dissipation under antegrade flow [40]. In our model the mitral, aortic, tricuspid and pulmonary valve are represented. We also introduce a single lumped venous valve in the systemic venous compartment (component $r_{ev}$).

The mechanical model is presented in Fig 2. It is a structurally extended version of our previous model [40] having a four-chamber heart. Ventricular interaction is not considered. The systemic circulation is divided into the upper body and lower body sub-systems, after Ursino [9]. The main differences between this model and that of Ursino [9] are:

1. a lack of an inertance, which improves numerical stability and therefore reduces computation time and memory requirements;
2. a venous valve $r_{eV}$ in the lower body compartment replacing a resistor, to enable free movement from the systemic circulation to the right heart and to prevent backflow;
3. an additional resistance $R_{Ao2}$ for the common iliac artery;
4. a non-linear compliance for the systemic venous compartment to more accurately describe blood pooling in highly elastic venous vessels;
5. a simplified pulmonary circulation. (The latter is not the focus of our study.)

**Fig 2:**
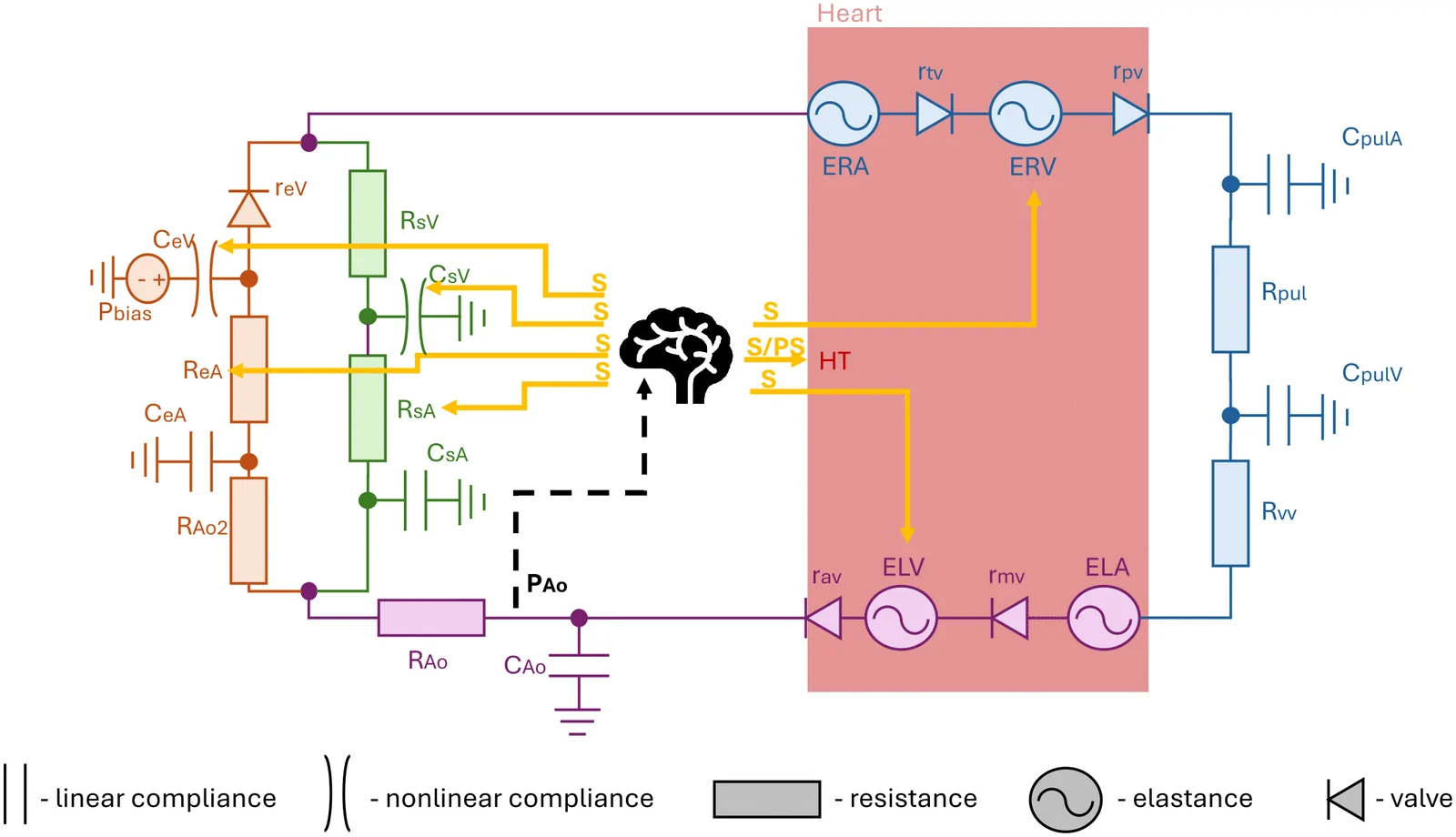
Schematic of our cardiovascular model architecture, with systemic (pink) and pulmonary (blue) circulations. In the systemic compartment upper body (green) and lower body (orange) circulations feature.

This architecture results in pressure, volume and flow variables in the following 11 compartments: left ventricle, aorta, upper body arteries, upper body veins, lower body arteries, lower body veins, right atrium, right ventricle, pulmonary arteries, pulmonary veins, and left atrium. A majority of cardiovascular orthostatic response models in the literature, e.g., [5] further divide the vasculature, into compartments (legs, arms, cerebral, etc.). Such models are adapted to resolve changes in blood distribution between vascular regions. The relative architectural simplicity of our model is a consequence of its setting- the estimation of available *global* relative SV, MAP and HR changes (in contradistinction to the quantification of absolute blood volume shifts between specific territories). In Fig 2, a signal $P_{bias}$ represents a source of external pressure (here, LBNP) acting on the *based* compartment. See section 2.4. An initial input parameterisation is taken from Ursino [9] and adjusted to match physiological limits for the atrial and ventricular pressure-volume (PV) loops of an adult woman. In the base state (without disturbance), the heart rate settles at around 55 beats per minute (bpm), and the cardiac output is approximately 5110 ml/min. Final values of all mechanical model input parameters are declared in Table 1.

### Baroreflex sub-model

In humans, the baroreflex system input is transduced from absolute pressures in the carotid sinus and aortic arch. To incorporate baroreflex effects on cardiovascular physiology, we use the model proposed by Ursino [9] without any modification, for its fidelity in representing the innervation of regulation – it describes the afferent and efferent processing of the neural signals, the advent of delays incurred in the central nervous system, the saturation of the spiking rates, and it includes effectors [9] on the parasympathetic (vagal) arc (for heart period) and sympathetic arc (for the heart period, left and right ventricular elastance, unstressed venous compartment volume and systemic vascular resistance). After Ursino, we denote by Δθ the regulated change to the mechanical base parameter $\theta_{base}$. For example, the regulation-induced, dynamic change to vascular resistance $R_{sA}$ is denoted $\Delta R_{sA}(t)$. The current value of the mechanical sub-model input parameter θ is then given by $\left(\theta_{base}+\Delta \theta (t)\right)$. Formally, Ursino’s sub-model of baroregulation may be designated *single input, multiple output* [43]. The base parameterisation of our baroregulation sub-model is taken from Table 3 in reference [9]; see also these authors’ previous work [40].

### Sub-model coupling

Our model is closed-loop. Mechanical sub-model parameters are modulated by the baroreflex effectors, while the input to the baroreflex is mechanical aortic pressure, which is compared to the regulation set-point. We assume that baroreceptors are located in the aortic arterial compartment, so the input to our regulation sub-model is the single, instantaneous value of aortic pressure; its outputs are the current values of system effectors. This is acceptable in the present setting, for a supine subject not subject to hydrostatic pressure changes. Effector increments (Δθ) to the mechanical sub-model’s passive compartment base parameterisations of unstressed volume (see section 2.4), resistance and compliance were computed from the dynamics declared by Ursino [9], then used to update the current effector value [9],. For example, vasoconstriction was simulated by modulating systemic arterial resistances $R_{sA}$, $R_{eA}$ and venous compliance values $C_{sV}$, and $C_{eV}$ with the current values of $R_{sA}+\Delta R_{sA}$, $R_{eA}+\Delta R_{eA}$, $C_{sV}+\Delta C_{sV}$, and $C_{eV}+\Delta C_{eV}$, respectively. In the case of the active (heart) compartment, elastance and heart period effectors were updated at the start of a beat, based upon changes continuously accumulated, over the preceding beat. Again, the regulation of unstressed volume requires special consideration; it serves as an example in section LBNP orthostatic challenge and unstressed volume regulation.

### LBNP orthostatic challenge and unstressed volume regulation

When recumbent, during a lower body negative pressure (LBNP) challenge, hydrostatic pressure differences can be ignored. Consequently, the input to the cardiovascular regulation system (carotid sinus total pressure) is identical, in our model, to the central aortic viscous pressure. During an LBNP challenge, negative pressure is applied to the legs. The effect of this perturbation is assumed to dominate in the lower body venous leg compartment, where an applied negative bias pressure causes blood to pool. The effect of regulation of unstressed volume is also considered in this compartment. In Ursino’s model [9] a sympathetic effector modulates unstressed volume in response to the pressure and flow changes that accompany blood pooling, with consequences for stressed volume. Any mechanically-driven increase in the stressed volume of the leg venous vessels is limited by the non-linear distensibility properties of vessel wall tissues. We model the non-linear unstressed venous volume response to a transmural pressure, $P_{t}$ (which is the sum of the computed viscous pressure and any applied external bias pressure associated with an orthostatic challenge) using a synthesis of the approaches of Heldt et al. [5] and Ursino [9]. After Heldt et al., the stressed volume is related to net compartmental transmural pressure $P_{t}$, by an associated compliance, as follows


$$\begin{array}{c} V=\alpha \tan^{-1}\left(\frac{C_{0}}{\alpha }P_{t}\right),\;\;C=\frac{dV}{dP_{t}}=\frac{C_{0}}{1+{\left(\frac{C_{0}P_{t}}{\alpha }\right)}^{2}}=\frac{C_{0}}{1+\tan^{2}\left(\frac{V}{\alpha }\right)}. \end{array}$$ (6)

Above, *C*_0_ is the compliance of the compartment at $P_{t}=0$ and the α depends on the maximum volume of the compartment, $V_{max}$


$$\begin{array}{c} \alpha =\frac{2V_{max}}{\pi }. \end{array}$$ (7)

It will be convenient to move between volume and pressure as independent variables in this section. Using the second of the above equations, we are able to change the variable of the non-linear compliance between *V* and *P* as desired.

Let us formulate the dynamics of the lower body venous compartment in the presence of non-linear compliance effects on the stressed volume, together with the baroregulation of the unstressed leg compartment volume. For a non-linear response, $C=C(P_{t})$, or, equivalently *C* = *C*(*V*). Differentiating on *t* and treating *C* as a mono-variate func*t*ion of *P* we have $\frac{d}{dt}\left(C(P_{t})P_{t}\right)=\frac{dV}{dt}=i$. Compartmental inflow *i* arises from two sources- the net mechanical flux at the boundary $i^{\prime }$, and the rate of change, under the action of volume regulation, of the unstressed volume $V_{u}$. Using the product rule, the chain rule, the definition of compliance, and a little algebra


$$\begin{array}{c} \frac{dP}{dt}=f(V)\left(i^{\prime }-\frac{d\Delta V_{u}}{dt}\right),\;f(V)=\frac{1}{C}{\left(P_{t}\frac{dC}{dV}+1\right)}^{-1}. \end{array}$$ (8)

Above, the negative sign arises because a reduction in the unstressed volume is a positive contribution to the stressed volume; further, the derivative of compliance is taken with respect to stressed volume. Equations 6 straightforwardly yield


$$\begin{array}{c} P=\frac{\alpha }{C_{0}}\tan\left(\frac{V}{\alpha }\right),\;\frac{dC}{dV}=-\frac{C_{0}}{\alpha }\sin\left(\frac{2V}{\alpha }\right). \end{array}$$ (9)

Using Equations 8 and 9 and trigonometric transformations we obtain an appropriate state-space form [43] for the dynamics of our regulated venous compartment, having non-linear compliance properties


$$\begin{array}{c} \frac{dP}{dt}=f(V)\left(i^{\prime }-\frac{dV_{u}}{dt}\right),\;f(V)=\frac{1}{C_{0}}\frac{\left(1+\tan^{2}\left(\frac{V}{\alpha }\right)\right)}{\left(1-\tan^{2}\left(\frac{V}{\alpha }\right)\right)}. \end{array}$$ (10)

Above, the term $\frac{dV_{u}}{dt}$ derives from our regulation model effectors [9]. Finally, we note that the transmural pressure has viscous (computed) and bias (assigned) contributions with $P_{t}(t)=P(t)-P_{bias}(t)$, that the viscous pressures and of course Ohm’s law determine the flow $i^{\prime }$, and that the bias pressure is negative; therefore we write our lower body compartment dynamics as follows


$$\begin{array}{c} \frac{dP}{dt}=f(V)\left(i^{\prime }-\frac{d\Delta V_{u}}{dt}\right)+\frac{dP_{bias}}{dt}. \end{array}$$ (11)

For our chosen orthostatic challenge, $P_{bias}$ is a continuous function of time, which we smooth to promote numerical stability.


$$\begin{array}{c} P_{bias}=\left\{ \begin{array}{cc} 0, & t\leq t_{start},\\ \frac{P_{max}}{2}\left(1-cos\left(\pi \frac{t-t_{start}}{t_{2}-t_{start}}\right)\right), & t_{start}<t\leq t_{2},\\ P_{max}, & t_{2}<t\leq t_{3},\\ \frac{Pmax}{2}\left(1+cos\left(\pi \frac{t-t_{3}}{t_{3}-t_{stop}}\right)\right), & t_{3}<t\leq t_{stop},\\ 0, & t>t_{stop}. \end{array}\right. \end{array}$$ (12)

In Equation (12), $t_{start}$, *t*_2_, *t*_3_ and $t_{stop}$ correspond, respectively, to *t*he absolu*t*e time of initialisation of the LBNP experiment, the end of the pressure-decreasing phase, the beginning of releasing the negative pressure, and the end of the experiment. Despite the trapezoid shape of the stimulus, slope-dependent overshoots are expected, similar to the square signal response described in Sturdy et al. [25] and Mahdi et al. [28].

### Model calibration

The pressures and volumes in our system with base input parameterisation, as well as comparisons between experimental results and model outputs, are presented in Fig 3. Clearly, the simulated time series of HR, MAP and SV vary from the recorded data. By a process of inverse model operation, one aims, in calibration, to assign model input parameters that allow the model to reproduce data from a specific patient or a cohort. From the mathematical perspective, calibration is often posed as the problem of finding the solution of *M* non-linear (probably) equations (one for each calibration target) in *M* unknown quantities (for the model input parameters we seek formally to identify). See Hose et al. [47] and the references therein. Issues of computational burden and numerical analysis notwithstanding, this approach can in principle calibrate a model to *M* measurements. Its computational cost is considerable and it is hard to achieve sufficient convergence (and therefore reliability of a solution). Moreover, problems arise when the number of observations is insufficient, e.g., when model outputs’ sensitivities and orthonormality [48,49] militate for a number of calibration parameters *N*:*N* > *M*. Under these circumstances, the problem is under-specified. Here, we advocate an adaptable alternative approach, viewing model calibration as an optimisation problem, conditioned by such properties of the model as its non-linearity. Operationally, our model calibration is the minimisation of a chosen scalar objective (loss) function, the value of which depends on all available experimental data.

**Fig 3:**
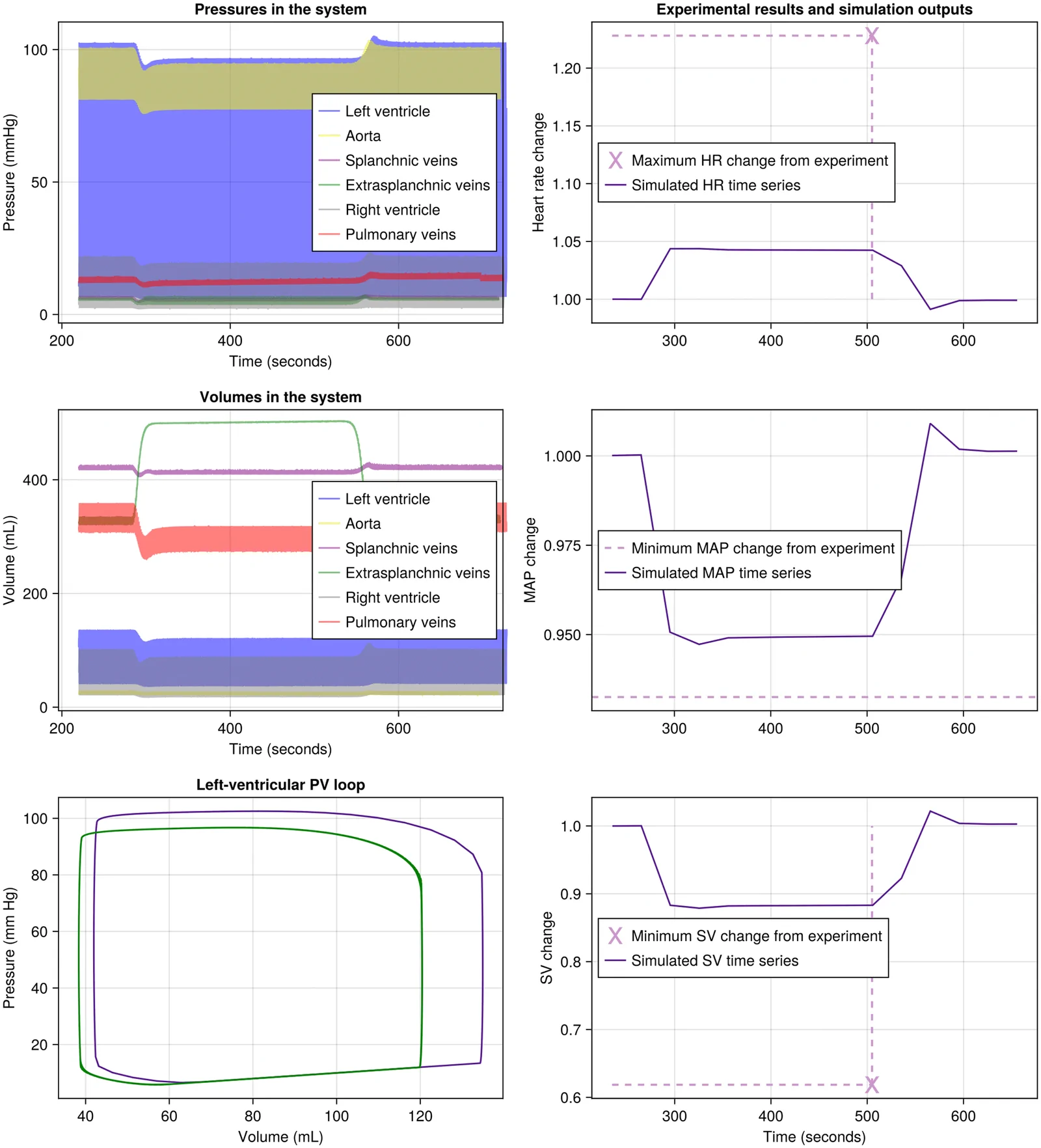
Closed-loop model responses, obtained with base parameterisation (Table 1 for mechanical parameters and [9] for baroreflex), when applying LBNP as described in section LBNP orthostatic challenge and unstressed volume regulation. Top left: system pressures over the LBNP orthostatic challenge. Middle left: system volumes over the challenge. Bottom left, left ventricular PV loop in the converged periodic state, at the start of the challenge. Top right: heart rate change, relative to its value at the start of the challenge, showing target experimental data [20]. Middle right: mean arterial pressure showing target experimental minimum value [20]. Bottom right: change in stroke volume, relative to its value at the start of the challenge, showing the showing target experimental minimum value [20]. The figures presented in this article were generated based on experimental data provided by the authors of the original study [20].

#### Character of our scalar loss function.

Our model is to be calibrated on cohort ensemble-averaged subject data, acquired over the duration of an orthostatic challenge. The base parameterisation for all model adaptations discussed in the following is declared in Table 1. For simplicity we chose to vary only baroreflex sub-model parameters. Typically, when calibrating, a converged periodic state, quantified by predicted arterial pressure is sought, matched to measurements which correspond to a converged periodic model state. The current investigation is more demanding. To address an entire orthostatic challenge, we must calibrate to unsteady-state experimental responses of several minutes’ duration. Mathematically, such a correspondence between time-distributed experimental data and a model prediction may be measured by a numerical estimate of a functional of i∈[1,N] computed model responses $f_{model,i}(t;\underset{―}{\Theta })$, as follows


$$\begin{array}{c} \Lambda \left[f_{model,i}(t;\underset{―}{\Theta }):i\in [1,N]\right]=\sum_{i=1}^{M}\int_{t_{start}}^{t_{end}}{\left\{f_{expt,i}(t)-g_{i}(f_{model,1}(t,\underset{―}{\Theta }),..,f_{model,N}(t,\underset{―}{\Theta }))\right\}}^{2}dt. \end{array}$$ (13)

Above, M≠N necessarily, $f_{expt,i}(t)$ is the i^th^ target experimental dataset (clinical metric), $g_{i}(\cdot )$ is a function mapping model outputs to the i^th^ clinical metric, the limits on the integral define the duration of the LBNP challenge (see next paragraph) and $\underset{―}{\Theta }$ denotes the model input parameter set. The latter must be specified before a numerical estimate of $\Lambda [f_{model,1}(t;\Theta ),...]$ can be obtained and are varied to minimise its value. To evaluate $\Lambda [f_{model,i}(t;\underset{―}{\Theta })]$, one must compute many cardiac cycles. To develop a qualitative understanding and reduce computation, we simplify the functional above, by (i) using derived properties of $f_{expt,i}(t)$ and (ii) interrogating an input parameter subset $\underset{―}{\theta }\in \underset{―}{\Theta }$, i.e., a parameter sub-space of reduced dimensionality.

#### Defining a loss function.

Our loss function is based on the experimental data provided by authors Høiseth and Hisdal. Their experiment is described in detail in reference [20]. Høiseth et al. studied 20 healthy adults. Supine subjects had their body below the iliac crest encased within an evacuated LBNP chamber and exposed to negative pressure of magnitude −60 mmHg for 4 minutes. Pain stimuli, induced by putting the subject’s hand in icy water were also applied simultaneously, but subject pain responses are beyond the present scope. Results were presented as ensemble-averaged time-series *changes* in stroke volume, heart rate, tissue oxygenation, mean arterial pressure, and cardiac output. Experiments were conducted for (i) LBNP alone, or (ii) LBNP + pain [20]. We focus on LBNP applied in isolation. A suitable loss, or objective, function was based on 8 observations, or derived subject metrics [20]:

1. maximum relative heart rate $\max(\Delta HR_{rel,exp})=1.2281$;
2. relative heart rate time response, over the challenge, has a triangular form;
3. minimum relative mean arterial pressure $\min(MAP_{exp})=0.9325$ (a 7% reduction);
4. the mean arterial pressure time series response over the challenge has a trapezoid form;
5. minimum relative stroke volume $\min(\Delta SV_{rel,exp})=0.6186$;
6. minimum relative stroke volume time series response over the challenge has a triangular form;
7. the time to achieve maximum relative heart rate $\Delta t_{HR_{exp}}=270$ seconds, from the start of the measurement;
8. the time to achieve minimum relative stroke volume $\Delta t_{SV_{exp}}=270$ seconds, from the start of measurement.

Our scalar loss function $L:{𝐑}^{N}\to {𝐑}^{1}$ which leverages the above, signature data properties 1,..,8 is defined as follows:


$$\begin{array}{cc} & L(\underset{―}{\theta })=G_{HR}+G_{MAP}+G_{SV}+T_{HR}+T_{SV},\\ & G_{HR}={\left(\frac{max(\Delta HR_{rel,sim})-max(\Delta HR_{rel,exp})}{max(\Delta HR_{rel,exp})}\right)}^{2},\\ & G_{MAP}={\left(\frac{min(MAP_{sim})-min(MAP_{exp})}{min(MAP_{exp})}\right)}^{2},\\ & G_{SV}={\left(\frac{min(\Delta SV_{rel,sim})-min(\Delta SV_{rel,exp})}{min(\Delta SV_{rel,exp})}\right)}^{2},\\ & T_{HR}={\left(\frac{\Delta t_{HR_{sim}}-\Delta t_{HR_{exp}}}{\Delta t_{HR_{exp}}}\right)}^{2},\\ & T_{SV}={\left(\frac{\Delta t_{SV_{sim}}-\Delta t_{SV_{exp}}}{\Delta t_{SV_{exp}}}\right)}^{2}. \end{array}$$ (14)

#### The tuning process.

Our tuning process seeks the global minimum $\min_{\underset{―}{\theta }}\left(L(\underset{―}{\theta })\right)$ in which *L*, as defined in Equation (14), is a scalar sum of squared difference terms. Like the parent functional $\Lambda \left[f_{model,i}(t;\underset{―}{\Theta }):i\in [1,N]\right]$, function $L(\underset{―}{\theta })$ is determined by experimental information and the vector of input parameters $\underset{―}{\theta }\in {\mathbb{R}}^{N}$, again with N≠M in general. Conceptually, we seek a minimum in a bounded response hyper-surface, in contradistinction to a zero. We deem this approach to be robust and – crucially – applicable in subspaces of appropriate dimension N≈M. This hyper-surface likely contains multiple, spatially separated minima, given our highly non-linear model. Moreover, our parameterisation, founded on Ursino’s work [9], is implicitly based on dogs, not humans. (To our knowledge, the ranges of baroreflex parameters for human subjects are not reported). Therefore, we assume the widest possible ranges of those model input parameters chosen for calibration. This increases the computational requirements for what we term, in what follows, the *global* optimisation, based on Differential Evolution. Consequently, it is challenging to achieve converged results when interrogating continuously variable input parameter search ranges within a practical timescale. This militates for an innovative approach to the optimisation problem.

Our strategy combines recursive global sensitivity analysis, orthogonality analysis (which is widely used in model reduction) together with changes of spatial resolution, which reduces the number of solution candidates, within a global optimisation designed to find a best solution (Fig 1).

#### Global sensitivity and orthogonality analysis.

We used Sobol’s method for the GSA to quantify the influence that model input parameters $\underset{―}{\theta }$ have on derived (scalar) model output *Y*. The total sensitivity indices of a model can be decomposed into partial sums of first-order and higher-order terms [50]:


$$\begin{array}{c} S_{T,i}(Y)=S_{1,i}(Y)+\sum_{i\neq j}S_{i,j}(Y)+\sum_{i\neq j\neq k}S_{i,j,k}(Y)+... \end{array}$$ (15)

First-order indices determine whether the separate input is influential on the output, while higher-order indices reveal the importance of interactions between the parameters. Based on Equation (15), higher-order elements can be calculated as a difference between the total-order $S_{T,i}(Y)$ and first order *S*_1,*i*_(*Y*) Sobol indices


$$\begin{array}{c} S_{T,i}(Y)=\frac{{𝔼}_{\theta_{i}^{c}}({𝕍}_{\theta }(Y|\theta_{i}^{c}))}{𝕍(Y)},\;S_{1,i}(Y)=\frac{{𝔼}_{\theta_{i}}\left({𝕍}_{\theta_{i}^{c}}(Y|\theta_{i})\right)}{𝕍(Y)},\;\theta_{i}^{c}=\underset{―}{\theta }/\theta_{i}. \end{array}$$ (16)

We used 58 mechanical and baroreflex parameters as model inputs. See Table 1 and references [9], and [51] respectively. Cardiac time constants (initial, peak-systolic, and end-systolic) were excluded from the analysis due to instability. All parameters were varied by ±15%. We recognise that this is only a small subregion of the whole search volume, but due to the computational requirements and instability associated with non-linearity of the model function, limiting the interrogated range in the sensitivity study was found to be necessary. We repeated the analysis in the best solution point identified in later steps, and compared the sensitivity pattern around both points to post-validate the results. Since GSA is a stage of the calibration process, its operation was focused on providing the most valuable information for the purpose of fitting the model to the data; therefore, we chose the components of the loss functions ($G_{HR},G_{MAP},G_{SV},T_{HR},T_{SV}$) and the loss function itself to be an output of the analysis. In so doing we hope to identify parameters that influence the fit in the desired directions. In addition to GSA, orthogonality analysis (OA) was performed on outputs’ relative sensitivity vectors, $\left\{S_{1,i}:i\in [1,N];i\in {\mathbb{Z}}^{+}\right\}$, the components of which were based on first-order Sobol indices, after Saxton et al.[48,49], in contradistinction to local sensitivities. The $S_{i,1}$ were used to identify parallel and orthogonal directions within the space spanned by model output sensitivity vectors, based upon the following measure of orthogonality [52]:


$$\begin{array}{c} d_{i^{\prime }i}=\sin\;[\cos^{-1}\;(\frac{S_{1,i^{\prime }}\cdot S_{1,i}}{\left\|S_{1,i^{\prime }}\|\;\|S_{1,i}\right\|})],\;i,i^{\prime }\in [1,..,N],\;i\in {\mathbb{Z}}^{+},\;d_{i^{\prime }i}\in [0,1]. \end{array}$$ (17)

Based on the combined results of OA and GSA, a subset of calibration parameters was identified. The same group of parameters (or their equivalents) was used to calibrate our previous, simple model. See Saxton et al., [48,49].

#### Differential evolution-based optimisation.

Differential evolution (DE) is a multi-point, derivative-free method of global function optimisation, in which the randomly generated population of candidate solutions is iteratively improved, to find the optimum [41]. For the *i*-th *j-*dimensional vector (individual) in the *g*-th generation of a population, iteration consists of three parts [41]:

1. Mutation: For each target vector ${\text{x}}_{i,g}$, base vector ${\text{x}}_{r0,g}$ and difference vectors (${\text{x}}_{r1,g}$ and ${\text{x}}_{r2,g}$) are chosen and combined to create a mutant ${\text{v}}_{i,g}$:

$$\begin{array}{c} {\text{v}}_{i,g}={\text{x}}_{r0,g}+F\cdot \left({\text{x}}_{r1,g}-{\text{x}}_{r2,g}\right), \end{array}$$ (18)

where $F\in {\mathbb{R}}^{+}$ is the evolution rate coefficient;

2. Crossover: From a discrete recombination of target vector ${\text{x}}_{i,g}$ and mutant vector ${\text{v}}_{i,g}$, create a trial random vector ${\text{u}}_{i,g}$ with components:

$$\begin{array}{c} u_{j,i,g}=\left\{ \begin{array}{cc} v_{j,i,g}, & \text{if }rand_{j}(0,1)\leq C,\\ x_{j,i,g}, & \text{if }rand_{j}(0,1)>C, \end{array}\right. \end{array}$$ (19)

where C∈[0,1] is crossover rate coefficient;

3. Selection: The value of *L* for trial vector ${\text{u}}_{i,g}$ and target vector ${\text{x}}_{i,g}$ is compared, and the vector with the smaller value *L* is assigned as the target vector, in next generation:

$$\begin{array}{c} {\text{x}}_{i,g+1}\left\{ \begin{array}{cc} {\text{u}}_{i,g}, & \text{if }f({\text{u}}_{i,g})\leq f({\text{x}}_{i,g}),\\ {\text{x}}_{i,g}, & \text{if }f({\text{u}}_{i,g})>f({\text{x}}_{i,g}). \end{array}\right. \end{array}$$ (20)

Iterations are repeated until a termination criterion, such as the maximum number of iterations, is met or, preferably, convergence is reached, i.e., the global minimum of the objective function is identified [41]. Here, the number of individual points (agents) is set to 120 and the termination criterion is the maximum number of search iterations (35k).

#### Change of search resolution.

DE optimisers search the whole parameter space ${\mathbb{R}}^{N}$ which challenges even High Performance Computing (HPC) infrastructure. As a redress, we add a step imposing a chosen discretisation within the search space, as declared in Table 2. The discretisation imposed on a particular input parameter reflects its base value and its clinical significance; put another way, the model is constrained to work on a curated mesh within input parameter space. Algorithmically, after a point is generated by the DE algorithm, its coordinates are rounded to the nearest mesh cell vertex and the objective function is recalculated. This practically-motivated step promotes convergence by relaxing accuracy but it introduces additional non-linearity and so generates convergence phenomena, which we seek to *integrate-out*, by repeating our calculation many times. As our best solution, we choose the point with the smallest objective function, identified during this process.

**Table 2:** Tabulation of the resolution and initial parameter ranges used in the calibration of the model input parameters to the orthostatic challenge data, using the Differential Evolution (DE) method.

Parameter	Lower Boundary	Upper Boundary	Resolution
$P_{n}$	85.00	95.00	1.00
$\tau_{HTs}$	1.00	60.00	1.00
$k_{es}$	0.05	0.15	0.01
$G_{Rep}$	0.50	3.00	0.01
$G_{Cvs}$	−270.00	−70.00	1.00
$G_{HTs}$	−0.60	−0.10	0.01

### Implementation

We used open-source Julia language-based utilities for our analysis and data visualisation. Delay Differential Equations were solved using the FSAL 5th order free-interpolants method [53], embedded in the DelayDiffEq.jl package [54,55], with absolute and relative tolerances set to 1e-7. The StaticArrays.jl package was used to reduce memory requirements for a single model run [56]. We also used QuasiMontecarlo.jl [57] and GlobalSensitivity.jl [58] packages to perform the GSA. Our global optimisation was performed using the DE implementation from the BlackBoxOptim.jl [59] package, with the interface of Optimization.jl [60] and Optim.jl [61] packages. Computations were performed on the Ares cluster at the PLGrid HPC Centre ACK Cyfronet AGH, equipped with Intel(R) Xeon(R) Platinum 8268 CPU with 48 cores and 192GB RAM on each node [62]. GSA entails significant computational expense as it requires multiple model evaluations. Our model is efficient from the perspective of its execution time (less than 5 seconds for the execution of a single model input parameter set) and its memory (less than 3.5GB, which allows one iteration to run on a single thread).

## Results

We present the results of the steps of the methodology shown in Fig 1. The purpose of the data we present is to support our overall objective: a calibration of our closed-loop model, to experimental cohort data, on a particular orthostatic challenge [20].

### Global sensitivity and orthogonality analysis

The values of Sobol indices obtained during initial GSA are presented in Fig 4. The most influential parameter is lower body venous compliance $C_{ev}$, which characterises the mechanical sub-model component to which the LBNP stimulus is predominately applied. It influences all extensive input parameters at first-order and timing parameters in interactions. Ventricular parameters are generally more influential than atrial (except for the maximum elastance of the right ventricle $ERV_{max}$). Unsurprisingly, most parameters of the systemic circulation (both upper body and lower body) are more influential than those of the pulmonary circulation. The most influential pulmonary parameter is pulmonary venous compliance, $C_{pulV}$. The influence of regulation gains and neural frequencies is also visible.

**Fig 4:**
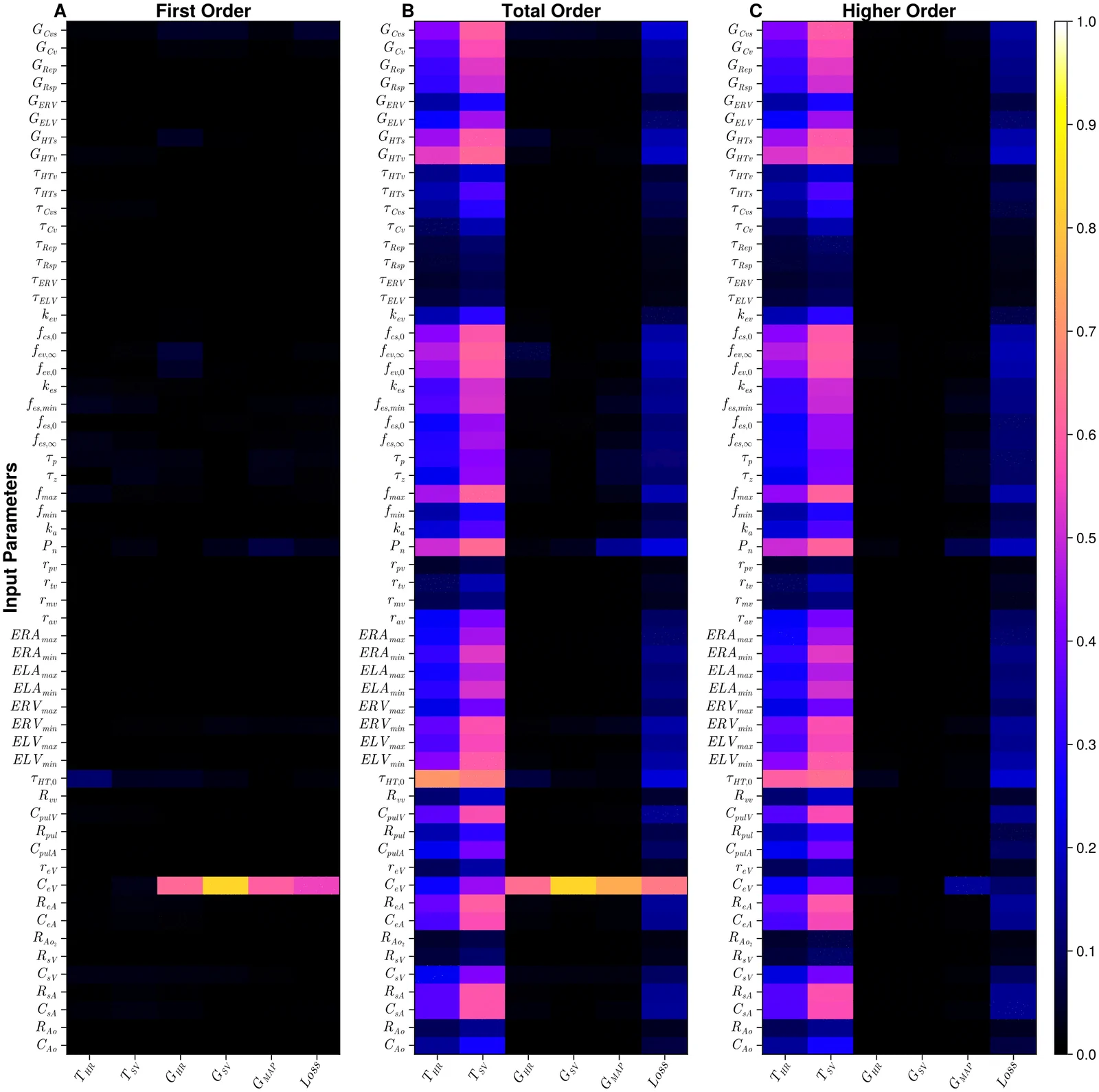
GSA I. A heatmap of the results of GSA on the coupled model’s loss function. This set of sensitivities is a guide to the calibration of the model. Input parameters for both regulation and mechanical sub-models are shown on the ordinate. Derived loss function terms, i.e., derived model outputs are shown on the abscissa, along with the overall loss function described in Equation (14). Panel (A) First-order Sobol indices, (B) total order and (C) higher-order. For these data, the model input parameters were varied by ±15 %.

The loss function term $G_{HR}$ is modulated by lower body venous compliance $C_{ev}$, sympathetic heart period regulation gain $G_{HTs}$ and vagal neural frequencies $f_{ev,0}$ and $f_{ev,\infty }$ (notation of Ursino [9]) in first-order. The magnitude of SV (represented here as $G_{SV}$) is primarily driven by lower body venous compliance $C_{ev}$, but the gain $G_{Cvs}$ that modulates the unstressed upper body volume is also influential. The magnitude of MAP ($G_{MAP}$) is modified by both higher-order and first-order interactions; here the highest values of Sobol indices are achieved for lower body venous compliance $C_{ev}$ and the baroreflex set-point.

When comparing the first-order indices (Fig 4 A) and the higher-order indices which were derived as the difference between the total-order and first-order indices and are shown in Fig 4C, it is clear that higher-order interactions dominate in shaping the objective function (especially the timing compartments, which are influenced by most parameters). This drastically complicates the choice of the calibration subset.

Orthogonality analysis of first-order Sobol indices (Fig 5) revealed that all time constants with the exception of the pair $\tau_{ERV}$ and $\tau_{HTv}$ influence the system in the same direction. They also show a high orthogonality coefficient with contractility gains and upper body and lower body parameters. All upper body and lower body parameters, except lower body venous compliance (to which the bias pressure is connected), show a similar effect on the objective function. Lower body venous compliance $C_{eV}$ acts similarly to both unstressed volume regulation gains $G_{Cv}$ and $G_{Cvs}$. Considering the heart, maximum values of ventricular elastance $ELV_{max}$ and $ERV_{max}$ have a closer influence to systemic parameters than the minimum elastance values. The situation is reversed when considering the atria. There is a high coefficient value between the resistance of the aortic valve $r_{av}$ and the rest of the systemic parameters, but it has a different influence direction with respect to the other heart valves, which all perturb the output in a similar direction. Contractility gains $G_{ELV}$ and $G_{ERV}$ show high orthogonality values relative to many systemic and baroreflex parameters, which is not true for the rest of baroreflex gains. In the upper right corner of the plot, we observe pairs of gains regulating the same properties of the vasculature (resistance, unstressed volume, contractility of the heart) and perturbing the model similarly, while heart period gains influence the system in different ways. The baroreflex set-point $P_{n}$ is an independent parameter, as it demonstrates a small orthogonality value with the majority of other parameters. The slope parameter of the sympathetic path $k_{es}$ and the time constants are characterised by a high orthogonality coefficient to the base value of the heart period $\tau_{HT,0}$, which was identified as an influential parameter in GSA.

**Fig 5:**
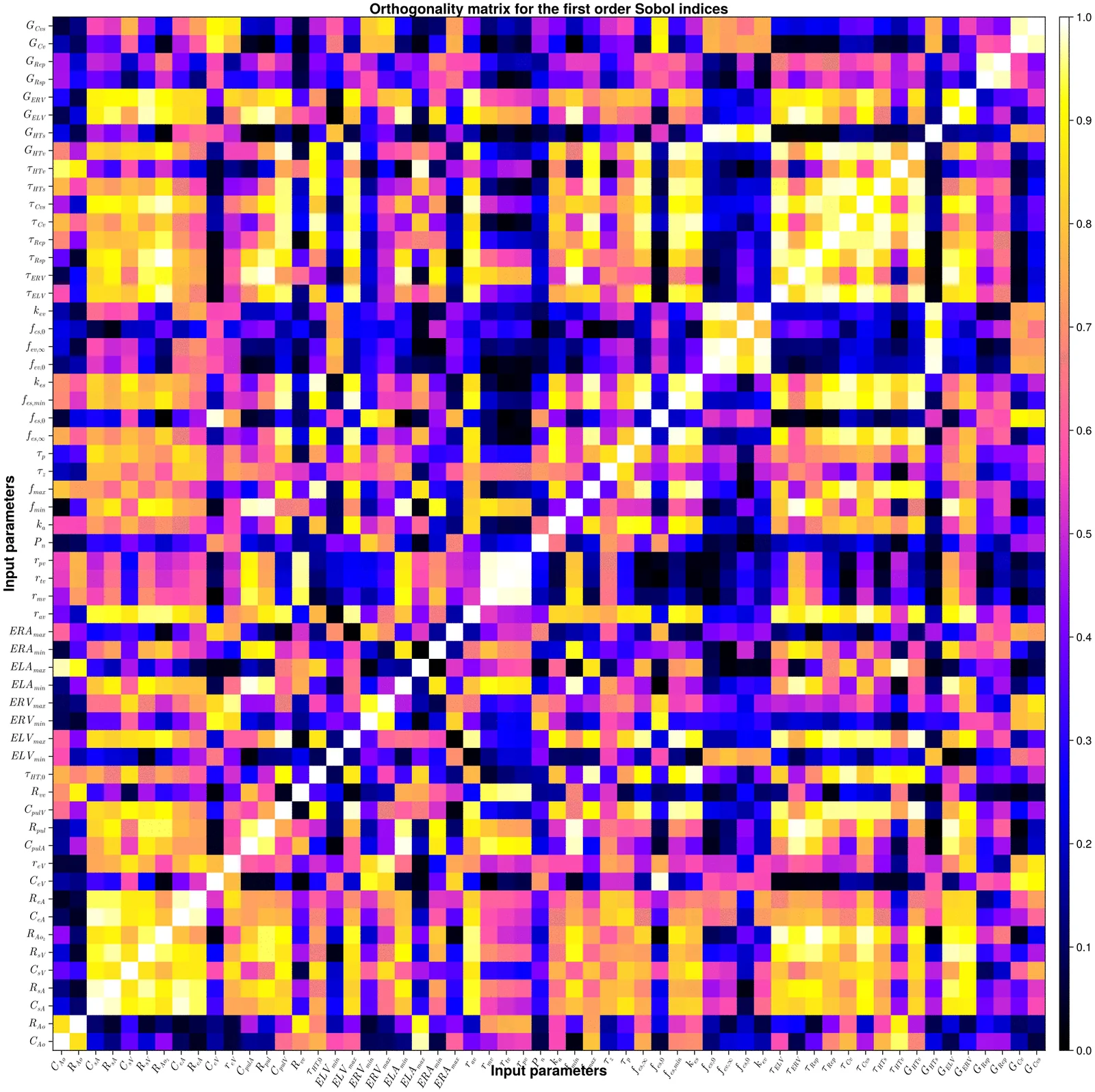
Orthogonality analysis of model sensitivity vectors. Sensitivity vector components were based on the absolute value of the first order Sobol indices (Fig 4). Dark colors indicate a small value of the dot product $d_{i,i^{\prime }}$ (Equation (17)), which indicates orthogonality (independence) of the effects on the outputs of the two parameters $\theta_{i}$ and $\theta_{i^{\prime }}$.

#### Choice of model input parameters for calibration.

LBNP experiments are designed to interrogate the baroreflex. Accordingly, we tune our closed-loop model LBNP-induced responses using parameters of our regulation sub-model. Furthermore, these parameters are our most uncertain, originating in small animal experiments [9]. The responses to a LBNP stimulus depend on the strength of the baroreflex feedback (innervation) and cardiovascular model properties, like venous compliance, as confirmed by our GSA. The parameter orthogonality matrix shows that it is possible to achieve similar effects using different parameter subsets. For example, changing unstressed volume gains ($G_{Cvs}$ or $G_{Cv}$) has a similar impact across model outputs as perturbing lower body compliance $C_{ev}$. Ultimately though, tuning using baroreflex parameters only is justified mainly by our model setting, i.e., the available data- we consider relative, cohort-averaged changes of SV, MAP and HR, with no information on a base state which would be necessary to credibly assess the mechanical model parameters. To select an effective subset of regulation sub-model parameters we consider the importance of the following five system properties, listed below with a brief supporting rationale:

1. cardiovascular system compliance: in LBNP, blood pooling and vessel stretching are causal;
2. cardiovascular system resistance: mediate important regulatory vasoconstriction effects on pressure;
3. heartbeat: primary baroreflex response and prominent in target LBNP data;
4. baroreflex response rate: influences dynamics of regulation responses;
5. baroreflex activity base level: influences dynamics regulation responses.

Considering (1),..,(5) alongside the results of GSA and OA, we select 6 regulation sub-model input parameters to effect model calibration, used throughout the following sections. They are listed, together with a justification of their inclusion, in Table 3. Finally, OA shows that certain pairs of regulation gains have (anti-) parallel action on the outputs; accordingly, we fix their ratio, using their base values [9]; in Ursino’s notation:

**Table 3:** The rationale for our selection of model input parameter subset for calibration. We nominate only regulation sub-model input parameters, note. Abbreviations used: OA – input parameter orthogonality analysis, GSA – global sensitivity analysis.

Parameter	Data source	Rationale
Venous unstressed volume regulation gain ($G_{Cvs}$)	GSA, OA	Influential in first-order and high-order; high orthogonality to the most influential parameter, $C_{eV}$; modulates compliance of the LBNP-perturbed vasculature.
Resistance gain ($G_{Rep}$)	OA	Only available parameter modulating system resistive properties; perturbs outputs in different ways compared with compliance and heart period gains providing additional flexibility.
Heart period gain ($G_{HTs}$)	GSA	Heart period gains are both influential with vagal gain most influential; small base value (of 0.09) leads to a conveniently small range (0.01–0.09) cf. sympathetic period gain (value −0.13, range (-∞, −0.13]
Sympathetic time constant for heart rate regulation ($\tau_{HTs}$)	GSA	A parameter controlling the time to reach heart rate maximum is needed; the most influential time constant is sympathetic time constant for heart period control.
Sensitivity of sympathetic activity ($k_{es}$)	OA	Perturbs outputs in similar way to base value of heart period, which is influential on both timing terms in the loss function, at first-order.
Baroreflex set-point ($P_{n}$)	GSA	Influential in first-order and high-order; identifies the regulation baseline.


$$\begin{array}{c} G_{Rsp}=1.31\times G_{Rep},\;G_{V_{u,ev}}=0.5\times G_{V_{u,sv}},\;\tau_{HTv}=0.75\times \tau_{HTs}. \end{array}$$ (21)

The last association above is enforced because it is impossible to regulate antagonistic heart period regulation with control of the sympathetic time constant alone.

### Calibration

Conducting the DE method 100 times, 2 unique solutions were obtained. In Table 4 we compare the initial and final values of the subset declared in (Tables 2 and 3), of the regulation sub-model parameters used in calibration. The initial values are based in *prior art* [9]. The value of the loss function for both solutions was identical to 7 decimal places, and significantly lower in both cases, compared to pre-optimisation values.

**Table 4:** Calibration results. A comparison of the initial values of the chosen calibration subset (Tables 2 and 3) of regulation sub-model parameters, which was based in *prior art* [9], with their values obtained by global optimisation. First column: parameters chosen for calibration; second column: initial values [9] of the chosen parameters, third column: first optimisation values; fourth column: second optimisation values.

Parameter	Initial value	Optimised value	
		Solution 1 (69/100 DE runs)	Solution 2 (31/100 DE runs)
loss	0.4982	0.00023898	0.00023892
$P_{n}$	92	94	95
$\tau_{HTs}$	2	44	43
$k_{es}$	0.0675	0.15	0.15
$G_{Rep}$	0.530	2.99	2.96
$G_{HTs}$	−0.13	−0.35	−0.34
$G_{Cvs}$	−265.4	−97	−96

The experimental LBNP response data used for calibration is cohort-averaged [20], temporally coarse-grained (in 30s intervals) and expressed in terms of signal change relative to a base physiological state taken at the start of the challenge. We chose to compare model and experimental data using the change factor for each interval which magnifies the difference between the two sets of data.

Modeled orthostatic challenge time-course responses for the calibration regulation sub-model parameter subset with the smallest loss function value are presented in Fig 6. There, we show model responses relative to experimentally measured SV, HR and MAP values. Clearly, this fit to data is much improved as a consequence of the operation of our workflow (compare Fig 3). Derived model response metrics are declared in Table 5. For both optimisation solutions, the model was found to simulate HR and SV changes with a small relative error. The MAP change is underestimated. Solution 1 provides a better fit to SV data, but for MAP and HR, solution 2 is better, especially in respect of MAP (for which solution 2 achieves a reduction in error exceeding a factor 4.5 compared with solution 1. Both optimisations 1 and 2 are in much better agreement with the experimental data than results obtained with our chosen base parameterisation [9].

**Fig 6:**
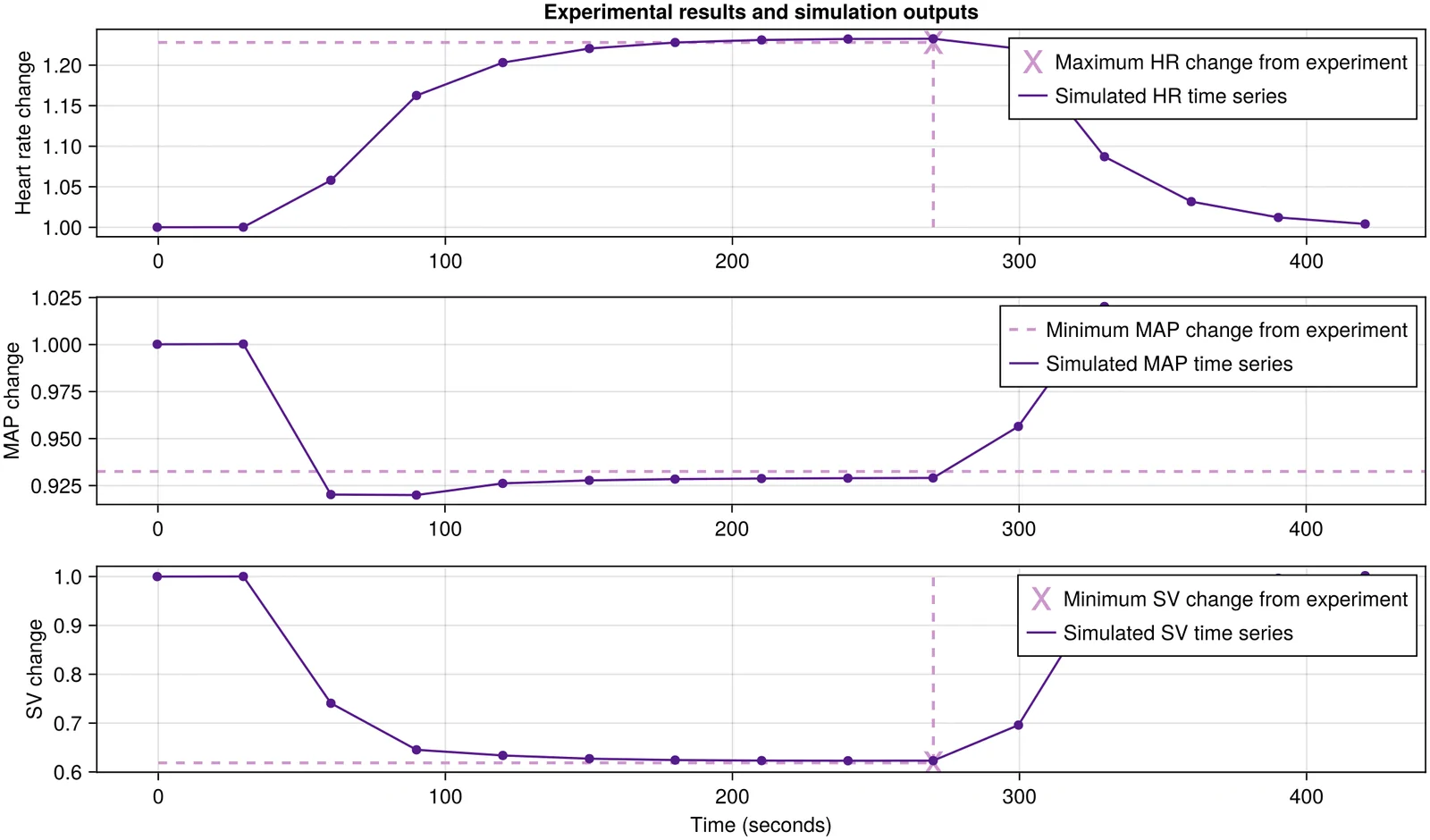
Time-course of modeled and experimental derived metrics, over the LBNP orthostatic challenge. Solid lines, changes in model outputs relative to the pre-challenge converged periodic state for solution 2. Broken lines, corresponding sampled experimental data [9]. Top panel, heart rate change. Middle panel MAP change. Bottom panel, SV change.

**Table 5:** Values of maximum relative HR, minimum MAP, minimum SV, and the time to achieve maximal HR and minimal SV during LBNP experiment. See Equation (14). Values calculated for experimental data and calibration solutions defined in Table 4.

Data source	Magnitude loss terms			Timing loss terms	
	$max(\Delta HR_{sim})$	$min(\Delta MAP_{sim})$	$min(\Delta SV_{sim})$	$\Delta t_{HR_{sim}}$	$\Delta t_{SV_{sim}}$
Experiment	1.2281	0.9325	0.6186	270	270
Base parameterisation	1.0435	0.9474	0.8788	180	180
Relative error (%)	−15.0313	1.5979	42.0627	−33.3333	−33.3333
Solution 1	1.2351	0.9196	0.6213	270	270
Relative error (%)	0.5700	−1.3834	0.4365	0.0000	0.0000
Solution 2	1.2333	0.9197	0.6224	270	270
Relative error (%)	0.4234	−1.3727	0.6142	0.0000	0.0000

In Fig 7 we present the model response with the best parameterisation, compared to the dynamics of the experimental changes of MAP, SV and HR described by Høiseth et al. [20], based on the data provided by these authors. Here, the reported value of the change is expressed relative to the value observed at the previous data point, to show the speed of fluctuations of physiological properties of the cardiovascular system during the experiment. The model performs well in simulating the middle part of the LBNP response, where it follows the experimental dynamics very well. Changes of SV, MAP and HR at the beginning of the experiment occur faster than in the experimental data. The model was not capable of representing the speed of SV fluctuations, when the stimulus was disabled.

**Fig 7:**
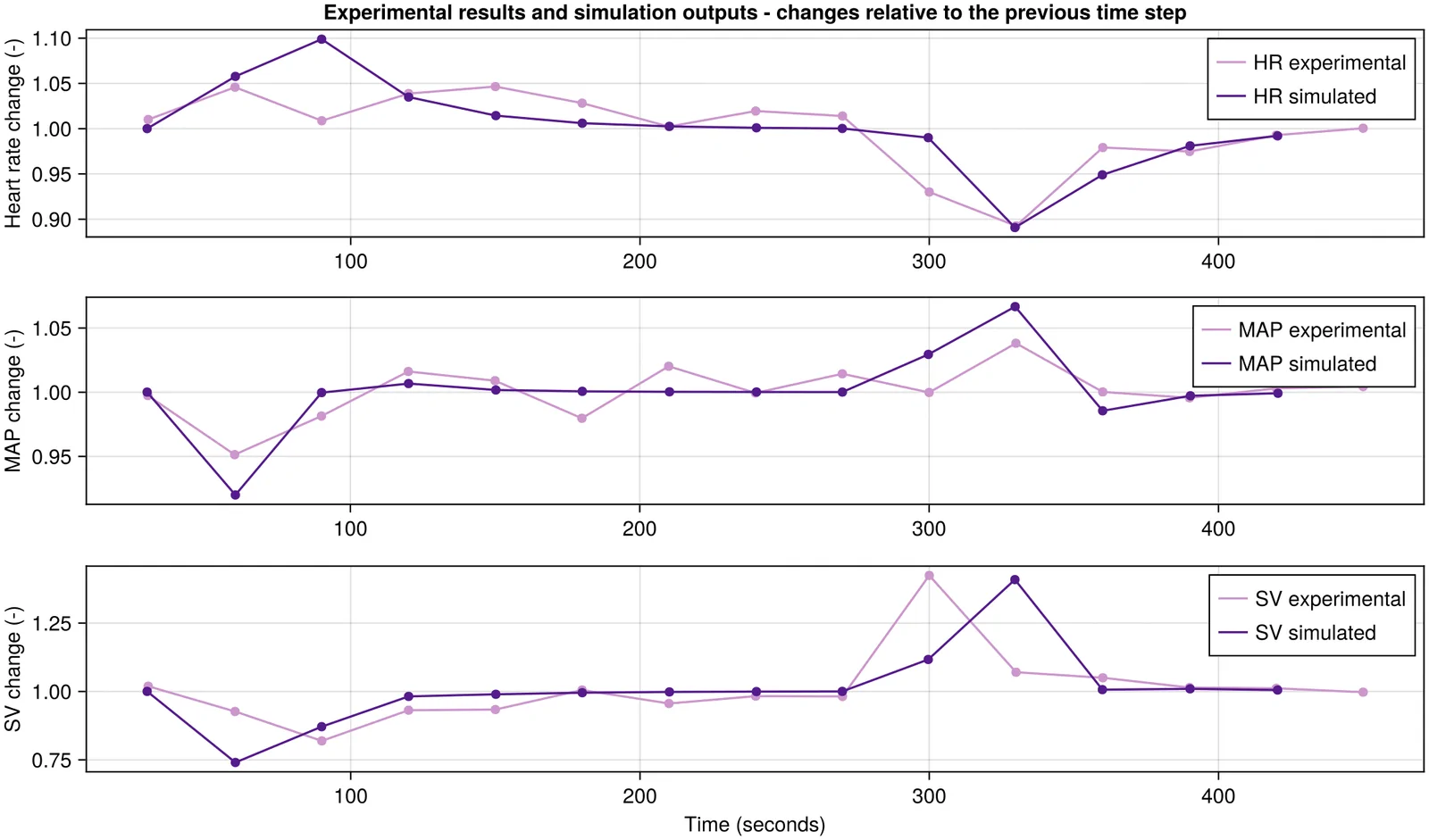
Model output and experimental data change factors defined for each 30s interval of the experimental data [20], and calculated for relative changes as follows. Top: relative change of HR. Middle: relative change in MAP. Bottom: relative change in SV. All changes are computed for the current time interval recorded on the abscissa, relative to the previous step. Solid lines are added to guide the eye only.

### Repeated GSA

GSA I, in sub-section 3.1, was conducted in a small volume of input parameter space, centered on a base state defined by our base parameterisation [9]. Therefore, here we conduct GSA II to quantify the variation of the sensitivity pattern. In Fig 8A–8C we present the results of GSA II, conducted ±15% about the best solution point. Comparing those results with data obtained by GSA I (Fig 4), one can see that key trends are preserved- specifically, the high non-linearity of the timing components of the loss function, the importance of lower body venous compliance $C_{eV}$ and the regulation set-point $P_{n}$. New influential parameters emerge in GSA II (Fig 8A–8C) – in particular the sympathetic heart rate regulation time constant $\tau_{HTs}$. One reason for the changes in sensitivities could be the increased range of the global sensitivity analysis, which results from the considerable change in certain calibration parameters. For example, $\tau_{HTs}$, for which the base value is 2 seconds, was analysed over the range [1.7, 2.3] in GSA I. The value of $\tau_{HTs}$ for the best solution is 43 seconds so for GSA II we have $\tau_{HTs}\in [36.55,49.45]$. Such an enlargement reduces the sampling density, significantly. To assess the impact of this effect, we performed a third GSA about the best solution point, but using input parameter bounds which were fixed to the values from GSA I, i.e., with parameter variations $\theta_{i}\in [\theta_{i}^{min}-\Delta \theta_{i0},\theta_{i}^{min}+\Delta \theta_{i0}]$ where $\Delta \theta_{i0}$ is the absolute change of parameter $\theta_{i}$, calculated as 15% from the base value of parameter *i* evaluated about our base parameterisation, $\theta_{i}^{\left(0\right)}$. Accordingly, we investigate the region (42.7;43.3) for $\tau_{HTs}$ and hence interrogate the same volume as that in GSA I. The result of GSA III is presented in Fig 8D–8F. Increased values of higher order Sobol indices are observed comparing our base parameterisation [9] (Fig 4) with the new parameterisation (Fig 8D–8F). The values of first-order indices decreased significantly. Upper body venous compliance $C_{sV}$ and the corresponding unstressed volume regulation gain $G_{Cvs}$ emerged as influential. The importance of sympathetic heart rate time constant $\tau_{HTs}$ is not visible here, in contrast to the case of GSA II (Fig 8A–8C).

**Fig 8:**
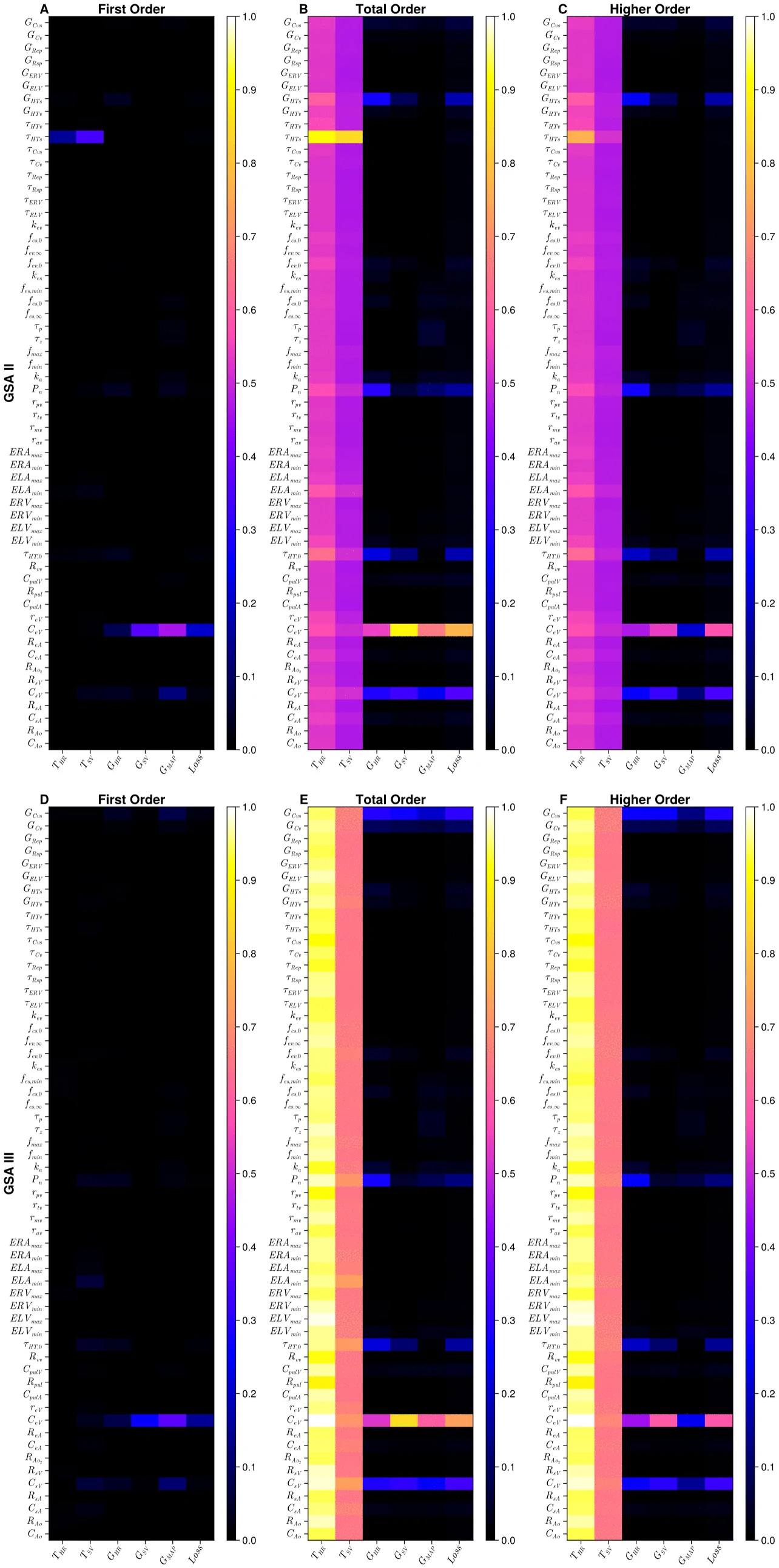
Comparison of GSA II and GSA III. Heatmaps of the results of the GSA on the coupled model’s loss function. Input parameters for both regulation and mechanical sub-models are shown on the ordinate. Derived loss function terms, i.e., derived model outputs are shown on the abscissa, along with the overall loss function. Within each row: left, first-order Sobol indices, middle total order and right higher-order. Top row: sensitivities about the calibrated base state for input parameter variation ±15%. Bottom row: absolute sensitivities about the calibrated base state for input parameter variation $\pm \Delta \theta_{i0}$.

## Discussion

The physiological adjustments presented above are in broad agreement with the *prior art* [5,17,19]. Heldt’s model has the greatest structural similarity to ours and predicts similar qualitative trend in HR and MAP response, albeit relative to different orthostatic challenge (i.e., HUT).

Centering a calibration strategy on the GSA of a curated loss function — i.e., a functional of protracted transient model outputs, rather than the outputs themselves — emerges as an effective approach, at least in the present setting. For an *a posteriori* justification of this observation, see sub-section below. It was clear from the outset that such a complex, nonlinear model as this, with many input parameters, would entail intractable parameter interactions and corresponding limits on the information available to guide model tuning. By employing a GSA based on a loss function, we can — again, in the present context — gain a clearer understanding of parameter influence *where it actually matters*, i.e., in relation to the properties of the target response. Of course, the latter must first be defined. Our solution to the curse of complexity might also be criticized for lacking portability, since the results of such a GSA apply only to the chosen loss function, and the assumptions about how the target response is characterised are inextricably embedded.

The complexity of the input parameter search space is exposed by multiple loss function minima. This finding militates for our recursive application of DE searches. That these repeated searches generate internally consistent information, as discussed in sub-section below, is very important. Without this re-assurance our approach is called into question. Domain knowledge might be expected to mitigate this concern but the cohort-averaged nature of the target data for our study and its counter-intuitive properties (specifically, long characteristic time responses) suggest that domain knowledge is no panacea.

### Global sensitivity and orthogonality analysis of the model

Consistent with previous studies [40], our GSA (Fig 4) exposes many higher-order input parameter interactions, reflective of the mathematical non-linearity and physiological complexity of the baroreflex regulation model of Ursino [9]. Non-linear effects gather around the timing terms of our loss function, indicating that the time to reach the extremum of the experimental response is influenced by a majority of input parameters. This hinders the identification of particular input parameters influencing those measurements. Comparing Sobol indices for baroreflex gains, arterial and venous vasoconstriction, and heart rate dominate over the regulation of cardiac contractility; this observation underscores the significance of these processes in the response to central hypovolemia and provides *a posteriori* justification for their inclusion in our tuning input set.

OA (Fig 5) shows all gains other than vagal ($G_{HTv}$) and contractility ($G_{ELV}$, $G_{ERV}$) are highly independent. Whilst physiologically plausible, this finding provides valuable information for the tuning process which requires a subset of independent and highly uncertain parameters. In the regulation of unstressed venous volume the gain ($G_{Cv}$) shows very similar orthogonalities to lower body venous compliance. Results presented in Fig 5 indicate also that the direction of influence of gains within a pair (resistance gains, unstressed volume gains, cardiac contractility gains) is similar. The exception is heart period regulation, in which the gains have highly independent effects.

Our study identified lower body venous compliance effects as an important factor in LBNP response. This is consistent with the experimental studies of Halliwill et al. [63], in which the authors describe the strong influence of leg compliance (a part of the lower body circulation) on venous pooling in healthy subjects. This result is expected: the main effect of LBNP is the pooling of blood to the lower regions of the body, which is limited by the structural properties of the vessels, such as their extensibility. In this work, the gain of unstressed volume regulation was used as a proxy of lower body venous compliance because it is hard to measure and the influence of both parameters on the loss function is similar. Finally, we remark on the increased importance of higher-order interactions in the neighborhood of the best solution and the decreased importance of varying parameters in isolation (Fig 8). The differences between the GSA II and GSA III results highlight the importance of changing ranges for the global analysis in non-linear models and underscore how important the way of defining parameter ranges is when the central point is moved significantly.

### Calibration of the model

A convergent result was made possible by changing the resolution of the search, by applying a rounding procedure after the DE random point selection. DE traditionally operates on real numbers. Here, we apply meshing in our input parameters hyperspace ${\mathbb{R}}^{6}$, pulling the random points to the nearest mesh node, thereby introducing additional non-linearity and risking spurious convergence. See Table 4. Accordingly, We advocate multiple DE executions, to decrease the probability of false results. Our two solutions differ but have a similar value of the objective function, possibly indicative of non-identifiability.

The values of the parameters found in the calibration process are very different from those reported by Ursino in Table 3 in reference [9]. We hypothesise that the reason is high uncertainty of the parameters and a base state collected mainly in animal studies [9]. A large discrepancy between the values reported before and the results obtained in our study calls into question the relevance of our GSA I, which covered ±15% value change from the initial parameterisation, which is (i) a significantly smaller volume than the search space of global optimisation; (ii) a region located far from the best identified solution. Quantifying Sobol indices in such wide range of parameters requires us to maintain fine sampling over the whole domain, which increases the computational requirements massively and increases the risk of falling into a numerically unstable region. To address the issue of misaligned sensitivity analysis scope, we repeated the analysis in a region concentrated around the best solution and compared the sensitivity patterns (Fig 8).

The most noticeable difference between the parametrisation that originates in the literature and the results of our study is in the value of the sympathetic time constant for HR regulation ($\tau_{HTs}$). We hypothesise that the reason might be a high non-linearity of a system visible in many higher order interactions affecting the timing parameters of the loss function. From visual inspection of experimental data for heart rate change, it is clear that the time constant of heart range change is much higher than reported in the literature. Here, in a model we included only baroreflex regulation, while during the experiment other mechanisms could influence the heartbeat. Orthogonality analysis showed that the sympathetic time constant for HR regulation ($\tau_{HTs}$) has a direction of influence similar to multiple parameters, including most of the parameters of the systemic circulation and other time constants. The value observed here may cover the cumulative effect of several small adjustments of the dependent parameters; therefore, it requires reinterpretation in the physiological context.

### Study limitations

Our focus is assessing whether the proposed methodology will improve accuracy in model fit to cohort level experimental data.

The architecture of our cardiovascular model is simpler than previous studies [5] applied to orthostatic response. Here, negative pressure was applied to an aggregated lower body compartment representing all vessels outside the upper body. While we reproduce target experimental measures correctly, our model does not resolve detailed blood volume shifts within the systemic circulation.

Our work was performed on averaged cohort data – individual differences between subjects are not resolved. Application of our workflow to patient-specific data, to study the variation of input parameters in a population, is the target of future work.

The experimental data are relative and do not provide enough information to evaluate absolute blood-volume distribution or base mechanical sub-model parameters, e.g., venous compliance of the lower body. Relative experimental data restricts interpretation of our tuned baroreflex sub-model parameters and how we select the tuning subset (recall, we fit baroreflex parameters only) – they are not unique, mechanical-system-independent estimates of physiological baroreflex response. We interpret them as values which, within the current model structure, represent the target data. It is impossible to differentiate between the effects of regulation and the effect of mechanical base state – that would require additional data.

With patient-specific data, fitting to a base mechanical state might be necessary, therefore our approach needs to be modified, if the purpose is, for example, to phenotype patients, based on parameter values (for example, by adding a supplementary round of optimisation).

The experimental time series used in this study was averaged over 30 second intervals, which might smooth some features of the experimental response to LBNP. Use of data with a finer temporal resolution may be more appropriate. Experimental data was collected in healthy subjects only.

The calibration parametrisation, which were significantly different from the base parameterisation, raises questions about appropriate sensitivity analysis and global optimisation ranges. Here, sensitivity analysis was performed within a smaller parameter volume than the optimisation, due to computational cost and model stability issues. There is a need for a computationally efficient approach to sensitivity quantification that is less susceptible to perturbations.

## Conclusion

We have performed the first calibration of a 58-parameter baroregulated 0D cardiovascular model to cohort-averaged lower-body negative pressure (LBNP) temporally resolved physiological responses [20], meeting the considerable challenge of fitting highly uncertain model input parameters, which requires coverage of a large volume of input parameter space ${\mathbb{R}}^{6}$, to experiments defining transient states of several minutes duration (in contradistinction to converged periodic states).

The technical challenge and computational expense of our calibration requires an innovative pipeline which leverages an underlying clinical rationale and utilises High Performance Computing. Specifically, traditional GSA is supplemented by a GSA approach based upon loss functional components as analysis outputs and input parameter orthogonality analysis; this expedient, together with physiological arguments, allows us to assess the utility of individual model input parameters in the calibration process, and to define an effective calibration set.

This work exposes general limitations in mathematical optimisation methods for stiff, non-linear physiological models with highly uncertain input parameters. Traditional DE operates on real numbers, i.e., float base types; very high uncertainty in input parameters necessitates wide search ranges, making it hard to achieve convergence with optimisers searching to high precision. At the potential cost of introducing additional non-linearity, our modified algorithm limits the search to a coarse mesh, which reduces the time needed to achieve convergence and simultaneously falls in line with practical clinical applications.

Our results suggest that translation of these models into clinical practice will be technically challenging and highlight the need for continued research.

## References

[1] Cardiovascular diseases. Accessed: 2024-12-04. https://www.who.int/health-topics/cardiovascular-diseases#tab=tab_1

[2] Costopoulos C, Timmins LH, Huang Y, Hung OY, Molony DS, Brown AJ, et al. Impact of combined plaque structural stress and wall shear stress on coronary plaque progression, regression, and changes in composition. Eur Heart J. 2019;40(18):1411–22. doi: 10.1093/eurheartj/ehz132 30907406 PMC6503452

[3] Morris PD, Narracott A, von Tengg-Kobligk H, Silva Soto DA, Hsiao S, Lungu A, et al. Computational fluid dynamics modelling in cardiovascular medicine. Heart. 2016;102(1):18–28. doi: 10.1136/heartjnl-2015-308044 26512019 PMC4717410

[4] Coorey G, Figtree GA, Fletcher DF, Snelson VJ, Vernon ST, Winlaw D, et al. The health digital twin to tackle cardiovascular disease-a review of an emerging interdisciplinary field. NPJ Digit Med. 2022;5(1):126. doi: 10.1038/s41746-022-00640-7 36028526 PMC9418270

[5] Heldt T. Computational models of cardiovascular response to orthostatic stress. Harvard University–MIT Division of Health Sciences and Technology; 2004.

[6] Norcliffe-Kaufmann L. Stress and the baroreflex. Auton Neurosci. 2022;238:102946. doi: 10.1016/j.autneu.2022.102946 35086020

[7] Kim J-K, Sala-Mercado JA, Rodriguez J, Scislo TJ, O’Leary DS. Arterial baroreflex alters strength and mechanisms of muscle metaboreflex during dynamic exercise. Am J Physiol Heart Circ Physiol. 2005;288(3):H1374-80. doi: 10.1152/ajpheart.01040.2004 15539416

[8] Fernandes LG, Müller LO, Feijóo RA, Blanco PJ. Closed-loop baroreflex model with biophysically detailed afferent pathway. Int J Numer Method Biomed Eng. 2024;40(9):e3849. doi: 10.1002/cnm.3849 39054666

[9] Ursino M. Interaction between carotid baroregulation and the pulsating heart: a mathematical model. Am J Physiol. 1998;275(5):H1733-47. doi: 10.1152/ajpheart.1998.275.5.H1733 9815081

[10] Laitinen T, Hartikainen J, Vanninen E, Niskanen L, Geelen G, Länsimies E. Age and gender dependency of baroreflex sensitivity in healthy subjects. J Appl Physiol (1985). 1998;84(2):576–83. doi: 10.1152/jappl.1998.84.2.576 9475868

[11] Gotoh TM, Fujiki N, Matsuda T, Gao S, Morita H. Roles of baroreflex and vestibulosympathetic reflex in controlling arterial blood pressure during gravitational stress in conscious rats. Am J Physiol Regul Integr Comp Physiol. 2004;286(1):R25-30. doi: 10.1152/ajpregu.00458.2003 14500268

[12] Jordan J, Ricci F, Hoffmann F, Hamrefors V, Fedorowski A. Orthostatic Hypertension. Hypertension. 2020;75(5):1151–8. doi: 10.1161/HYPERTENSIONAHA.120.14340 32223382

[13] Goswami N, Blaber AP, Hinghofer-Szalkay H, Convertino VA. Lower Body Negative Pressure: Physiological Effects, Applications, and Implementation. Physiol Rev. 2019;99(1):807–51. doi: 10.1152/physrev.00006.2018 30540225

[14] Smietanowski M, Cudnoch Jedrzejewska A, Dziuda L. Application of orthostatic test and lower body negative pressure in investigation of circulatory reflexes in humans. Folia Cardiol. 2015;10:283–7. doi: 10.5603/FC.2015.0050

[15] Morris PD, Anderton RA, Marshall-Goebel K, Britton JK, Lee SMC, Smith NP, et al. Computational modelling of cardiovascular pathophysiology to risk stratify commercial spaceflight. Nat Rev Cardiol. 2024;21(10):667–81. doi: 10.1038/s41569-024-01047-5 39030270

[16] Croston RC, Fitzjerrel DG. Cardiovascular model for the simulation of exercise, lower body negative pressure, and tilt experiments. Modeling and simulation Volume 5 - Fifth Annual Pittsburgh Conference, Conference Proceedings, ID 19750040674. 1974.

[17] Melchior FM, Srinivasan RS, Thullier PH, Clère JM, New Collective Author. Simulation of cardiovascular response to lower body negative pressure from 0 to -40 mmHg. J Appl Physiol (1985). 1994;77(2):630–40. doi: 10.1152/jappl.1994.77.2.630 8002508

[18] Hinghofer-Szalkay H. Gravity, the hydrostatic indifference concept and the cardiovascular system. Eur J Appl Physiol. 2011;111(2):163–74. doi: 10.1007/s00421-010-1646-9 20857139

[19] Fois M, Maule SV, Giudici M, Valente M, Ridolfi L, Scarsoglio S. Cardiovascular response to posture changes: multiscale modeling and in vivo validation during head-up tilt. Front Physiol. 2022;13. doi: 10.3389/fphys.2022.826989 PMC8892183 35250630

[20] Høiseth LØ, Hisdal J, Hoff IE, Hagen OA, Landsverk SA, Kirkebøen KA. Tissue oxygen saturation and finger perfusion index in central hypovolemia: influence of pain. Crit Care Med. 2015;43(4):747–56. doi: 10.1097/CCM.0000000000000766 25513787

[21] Lim E, Chan GSH, Dokos S, Ng SC, Latif LA, Vandenberghe S, et al. A cardiovascular mathematical model of graded head-up tilt. PLoS One. 2013;8(10):e77357. doi: 10.1371/journal.pone.0077357 24204817 PMC3812216

[22] Jennekens W, Dat M, Bovendeerd PHM, Wijn PFF, Andriessen P. Validation of a preterm infant cardiovascular system model under baroreflex control with heart rate and blood pressure data. Annu Int Conf IEEE Eng Med Biol Soc. 2011;2011:896–9. doi: 10.1109/IEMBS.2011.6090200 22254455

[23] Gee MM, Lenhoff AM, Schwaber JS, Ogunnaike BA, Vadigepalli R. Closed-loop modeling of central and intrinsic cardiac nervous system circuits underlying cardiovascular control. AIChE J. 2023;69(4):e18033. doi: 10.1002/aic.18033 37250861 PMC10211393

[24] Schild J. A mathematical model of the afferent stage of the mammalian baroreceptor reflex. Houston (TX): Rice University; 1994.

[25] Sturdy J, Ottesen JT, Olufsen MS. Modeling the differentiation of A- and C-type baroreceptor firing patterns. J Comput Neurosci. 2017;42(1):11–30. doi: 10.1007/s10827-016-0624-6 27704337 PMC5253111

[26] Hodgkin AL, Huxley AF. A quantitative description of membrane current and its application to conduction and excitation in nerve. J Physiol. 1952;117(4):500–44. doi: 10.1113/jphysiol.1952.sp004764 12991237 PMC1392413

[27] Danielsen M, Ottesen J. A baroreceptor model. In: Ottesen J, Olufsen M, Larsen J, editors. No. 9 in S I A M Monographs on Mathematical Modeling and Computation. Society for Industrial and Applied Mathematics; 2004.

[28] Mahdi A, Sturdy J, Ottesen JT, Olufsen MS. Modeling the afferent dynamics of the baroreflex control system. PLoS Comput Biol. 2013;9(12):e1003384. doi: 10.1371/journal.pcbi.1003384 24348231 PMC3861044

[29] Sarmiento CA, Hernández AM, Serna LY, Mañanas MÁ. An integrated mathematical model of the cardiovascular and respiratory response to exercise: model-building and comparison with reported models. Am J Physiol Heart Circ Physiol. 2021;320(4):H1235–60. doi: 10.1152/ajpheart.00074.2020 33416450

[30] Van Willigen BG, van der Hout-van der Jagt MB, Huberts W, van de Vosse FN. A Multiscale Mathematical Model for Fetal Gas Transport and Regulatory Systems During Second Half of Pregnancy. Int J Numer Method Biomed Eng. 2025;41(1):e3881. doi: 10.1002/cnm.3881 39625422 PMC11661441

[31] Ottesen JT, Mehlsen J, Olufsen MS. Structural correlation method for model reduction and practical estimation of patient specific parameters illustrated on heart rate regulation. Math Biosci. 2014;257:50–9. doi: 10.1016/j.mbs.2014.07.003 25050793 PMC4252605

[32] Olufsen MS, Tran HT, Ottesen JT, Research Experiences for Undergraduates Program, Lipsitz LA, Novak V. Modeling baroreflex regulation of heart rate during orthostatic stress. Am J Physiol Regul Integr Comp Physiol. 2006;291(5):R1355-68. doi: 10.1152/ajpregu.00205.2006 16793939

[33] Kosinski SA, Carlson BE, Hummel SL, Brook RD, Beard DA. Computational model-based assessment of baroreflex function from response to Valsalva maneuver. J Appl Physiol (1985). 2018;125(6):1944–67. doi: 10.1152/japplphysiol.00095.2018 30236047 PMC6442666

[34] Olufsen MS, Ottesen JT. A practical approach to parameter estimation applied to model predicting heart rate regulation. J Math Biol. 2012;67(1):39–68. doi: 10.1007/s00285-012-0535-8 22588357 PMC3526689

[35] Golub GH, Van Loan CF. Matrix Computations. Johns Hopkins Studies in the Mathematical Sciences. Johns Hopkins University Press; 1996. Available from: https://books.google.pl/books?id=mlOa7wPX6OYC

[36] Colunga AL, Colebank MJ, REU Program, Olufsen MS. Parameter inference in a computational model of haemodynamics in pulmonary hypertension. J R Soc Interface. 2023;20(200):20220735. doi: 10.1098/rsif.2022.0735 36854380 PMC9974303

[37] Taylor-LaPole AM, Paun LM, Lior D, Weigand JD, Puelz C, Olufsen MS. Parameter selection and optimization of a computational network model of blood flow in single-ventricle patients. J R Soc Interface. 2025;22(223):20240663. doi: 10.1098/rsif.2024.0663 40013336 PMC11865955

[38] Saxton H, Xu X, Halliday I, Schenkel T. New perspectives on sensitivity and identifiability analysis using the unscented kalman filter. 2023. 10.48550/arXiv.2306.15710

[39] Matzuka B, Mehlsen J, Tran H, Olufsen MS. Using Kalman Filtering to Predict Time-Varying Parameters in a Model Predicting Baroreflex Regulation During Head-Up Tilt. IEEE Trans Biomed Eng. 2015;62(8):1992–2000. doi: 10.1109/TBME.2015.2409211 25769142

[40] Tlałka K, Saxton H, Halliday I, Xu X, Narracott A, Taylor D, et al. Sensitivity analysis of closed-loop one-chamber and four-chamber models with baroreflex. PLoS Comput Biol. 2024;20(12):e1012377. doi: 10.1371/journal.pcbi.1012377 39715272 PMC11706439

[41] Price KV. Differential evolution: a practical approach to global optimization. Natural computing series. London, England: Springer; 2004.

[42] Shi Y, Lawford P, Hose R. Review of zero-D and 1-D models of blood flow in the cardiovascular system. Biomed Eng Online. 2011;10:33. doi: 10.1186/1475-925X-10-33 21521508 PMC3103466

[43] Jacobs OLR. Introduction to control theory. London, England: Oxford University Press; 1974.

[44] Spiegel R. Stressed vs. unstressed volume and its relevance to critical care practitioners. Clin Exp Emerg Med. 2016;3(1):52–4. doi: 10.15441/ceem.16.128 27752616 PMC5051617

[45] Suga H, Sagawa K. Instantaneous pressure-volume relationships and their ratio in the excised, supported canine left ventricle. Circ Res. 1974;35(1):117–26. doi: 10.1161/01.res.35.1.117 4841253

[46] Korakianitis T, Shi Y. Numerical simulation of cardiovascular dynamics with healthy and diseased heart valves. J Biomech. 2006;39(11):1964–82. doi: 10.1016/j.jbiomech.2005.06.016 16140309

[47] Hose DR, Lawford PV, Huberts W, Hellevik LR, Omholt SW, van de Vosse FN. Cardiovascular models for personalised medicine: Where now and where next? Med Eng Phys. 2019;72:38–48. doi: 10.1016/j.medengphy.2019.08.007 31554575

[48] Saxton H, Xu X, Schenkel T, Clayton RH, Halliday I. Convergence, sampling and total order estimator effects on parameter orthogonality in global sensitivity analysis. PLoS Comput Biol. 2024;20(7):e1011946. doi: 10.1371/journal.pcbi.1011946 39018334 PMC11285933

[49] Saxton H, Schenkel T, Halliday I, Xu X. Personalised parameter estimation of the cardiovascular system: Leveraging data assimilation and sensitivity analysis. J Comput Sci. 2023;74:102158. doi: 10.1016/j.jocs.2023.102158

[50] Saltelli AA, Ratto M, Andres T, Campolongo F, Cariboni J, Gatelli D, et al. Global sensitivity analysis. Hoboken (NJ): Wiley-Blackwell; 2008.

[51] Ursino M, Antonucci M, Belardinelli E. Role of active changes in venous capacity by the carotid baroreflex: analysis with a mathematical model. Am J Physiol. 1994;267(6 Pt 2):H2531–46. doi: 10.1152/ajpheart.1994.267.6.H2531 7810748

[52] Tlałka K, Saxton H, Halliday I, Xu X, Taylor D, Narracott A, et al. Local Sensitivity Analysis of a Closed-Loop in Silico Model of the Human Baroregulation. In: Franco L, de Mulatier C, Paszynski M, Krzhizhanovskaya VV, Dongarra JJ, Sloot PMA, editors. Computational Science – ICCS 2024. Cham: Springer Nature Switzerland; 2024. p. 173–87.

[53] Tsitouras Ch. Runge–Kutta pairs of order 5(4) satisfying only the first column simplifying assumption. Comput Mathemat Appl. 2011;62(2):770–5. doi: 10.1016/j.camwa.2011.06.002

[54] Rackauckas C, Nie Q. DifferentialEquations.jl – A Performant and Feature-Rich Ecosystem for Solving Differential Equations in Julia. JORS. 2017;5(1):15. doi: 10.5334/jors.151

[55] Widmann D, Rackauckas C. DelayDiffEq: Generating Delay Differential Equation Solvers via Recursive Embedding of Ordinary Differential Equation Solvers. arXiv preprint arXiv:220812879. 2022.

[56] StaticArrays.jl. Package site. Available from: https://github.com/JuliaArrays/StaticArrays.jl

[57] QuasiMonteCarlo.jl. Package site. Available from: https://docs.sciml.ai/QuasiMonteCarlo/stable/

[58] Dixit VK, Rackauckas C. GlobalSensitivity.jl: Performant and Parallel Global Sensitivity Analysis with Julia. JOSS. 2022;7(76):4561. doi: 10.21105/joss.04561

[59] BlackBoxOptim.jl. Package site. Available from: https://github.com/robertfeldt/BlackBoxOptim.jl

[60] Dixit VK, Rackauckas C. Optimization.jl: A Unified Optimization Package. Zenodo; 2023. Available from: doi: 10.5281/zenodo.7738525

[61] Mogensen PK, White JM, Riseth AN, Holy T, Lubin M, Christof, et al. JuliaNLSolvers/Optim.jl: v1.13.2 (docs). Zenodo; 2025. Available from: doi: 10.5281/zenodo.15649599

[62] HPC resources documentation at Academic Computer Centre Cyfronet AGH. Ares Supercomputer; Accessed: 2024-12-18. Available from: https://docs.hpc.cyfronet.pl/supercomputers/ares/

[63] Halliwill JR, Lawler LA, Eickhoff TJ, Joyner MJ, Mulvagh SL. Reflex responses to regional venous pooling during lower body negative pressure in humans. J Appl Physiol (1985). 1998;84(2):454–8. doi: 10.1152/jappl.1998.84.2.454 9475852

